# Gut microbiota 16S rRNA profiling with plasma and urine metabolomics in vestibular migraine

**DOI:** 10.3389/fneur.2026.1722220

**Published:** 2026-03-19

**Authors:** Qijun Yu, Yanan Huang, Yanxue Ren, Changman Zhang, Jinghan Lin, Shijiao Chen, Hongyan Li, Changchang Ying, Zhihui Zhu, Qingling Zhai, Tingting Sun, Yonghui Pan

**Affiliations:** 1 Department of Neurology, The First Affiliated Hospital of Harbin Medical University, Harbin, China; 2 Department of Neurology, Beijing Nuclear Industry Hospital, Beijing, China

**Keywords:** 16S rRNA, migraine, plasma metabolomics, urine metabolomics, vestibular migraine

## Abstract

**Background:**

Vestibular migraine (VM) pathophysiology remains unclear, with research often extrapolating from migraine (M) studies. The microbiota-gut-brain axis represents a novel avenue for exploring VM mechanisms and treatments. This study aimed to compare gut microbiota, plasma and urine metabolome alterations among VM patients, M patients, and healthy controls (Hcs).

**Methods:**

A cross-sectional study recruited 15 VM patients, 15 M patients, and 15 age-/gender-matched Hcs (April–September 2024) from a tertiary hospital. Final analysis included 10 VM, 15 M, and 15 Hc participants. All underwent fecal 16S rRNA gene sequencing, plasma and urine metabolomics. Demographic and clinical data were collected.

**Results:**

Gut microbiota alpha/beta diversity showed no significant inter-group differences. At the phylum level, Verrucomicrobiota and Chloroflexi were reduced in VM vs. M. Genus-level analysis revealed trends (e.g., decreased *Akkermansia*, increased *Ruminococcus gnavus* group in VM) and LEfSe (Linear Discriminant Analysis Effect Size) identified several genera (e.g., *Latilactobacillus, Parvimonas*) with higher relative abundance in VM. Plasma metabolomics identified numerous differential metabolites; bioinformatics implicated amino acid metabolism pathways, with pyruvate as a key differential metabolite. Urine metabolomics and bioinformatics strongly associated differential metabolites with tyrosine metabolism and norepinephrine (NE).

**Conclusions:**

In this exploratory multi-omics study, VM patients showed distinct gut microbiota composition compared to M and HC, particularly at the genus level. Plasma metabolomics revealed alterations in pyruvate and amino acid metabolism pathways, suggesting possible energy metabolism disturbances in VM. Urine metabolomics highlighted the tyrosine metabolism pathway, with norepinephrine emerging as a metabolite of interest. These preliminary findings point to potential involvement of gut dysbiosis, metabolic perturbations, and neurotransmitter-related pathways in VM pathophysiology, providing a foundation for hypothesis-driven research. Given the exploratory nature of this study, these observations require validation in larger cohorts.

## 1 Introduction

VM is one of the most prevalent vestibular disorders, with an estimated 1-year prevalence of 2.7% in the general population (1) and 1.07% in Asians (2). Among neurology outpatients, the proportions of definite vestibular migraine (dVM) and probable vestibular migraine (pVM) are 4.6% and 1.1%, respectively (3). VM predominantly affects middle-aged women (4–7). Its core symptoms involve various forms of vertigo (vestibular symptoms), often accompanied by a history of migraine or migraine-like features during attacks, including visual aura, photophobia, or phonophobia (8). Moderate to severe vestibular symptoms significantly impair patients' daily life, work, and study. Reports indicate that VM patients have a higher rate of work absenteeism or school absence (60%) compared to patients with other vertigo or balance disorders (51%, *P* < 0.001). Furthermore, the proportion experiencing falls in the past 5 years (52.2%) is significantly higher than in the general population (17.0%, *P* < 0.001) or in patients with dizziness (43.7%, *P* < 0.01) (1). Additionally, Akdal et al. (9) identified cognitive impairment in VM patients, with another study (10) suggesting that the severity of dizziness may influence the degree of cognitive impairment.

The pathophysiological mechanisms underlying VM remain unclear, and no specific treatment guidelines exist. Despite increasing clinical research and the release of relevant expert consensus in China (11), current understanding and therapeutic approaches for VM are largely extrapolated from migraine. Proposed theories for VM pathogenesis include: a lowered trigeminovascular activation threshold; cortical spreading depression affecting vestibular processing centers in the brain; inherited ion channel defects; labyrinthine vasospasm; sterile inflammation affecting inner ear structures; and dysfunction in central vestibular pathways modulated by the dorsal raphe nucleus and locus coeruleus, potentially influencing cognitive behavior (12–18).

The growing recognition of the microbiota-gut-brain axis has spurred interest in the relationship between gut microbiota and neuropsychiatric disorders. Numerous studies have revealed gut dysbiosis, increased intestinal permeability, elevated pro-inflammatory cytokines, reduced levels of beneficial short-chain fatty acids (SCFAs) with anti-inflammatory properties, and dysregulated metabolism (e.g., tryptophan) in migraine patients. These alterations may contribute to migraine pathophysiology, potentially via neuroinflammation. Interventions like probiotics have shown promise in alleviating some migraine symptoms.

However, no studies to date have investigated the association between VM and gut microbiota. This observational study aims to compare the gut microbiota (via fecal 16S rRNA sequencing), plasma metabolome, and urine metabolome among VM patients, migraine (M) patients, and healthy controls (Hcs). We seek to identify differential gut bacteria and metabolites, and utilize bioinformatics to explore potential microbe-metabolite pathways. This foundational work aims to inform future research into the pathophysiology and therapeutic strategies for VM.

## 2 Materials and methods

This study was approved by the Ethics Committee of the First Affiliated Hospital of Harbin Medical University (Approval No. IRB-AF/SC-12/03.0). All participants provided written informed consent.

### 2.1 Participants

From April 2024 to September 2024, 15 patients with VM and 15 patients with migraine were recruited from the Dizziness and Vertigo Clinic, Headache Clinic, and Neurology Outpatient Department, as well as inpatients from Fourth Neurology Ward of the First Affiliated Hospital of Harbin Medical University. Additionally, 15 healthy controls were recruited from the family members of patients attending the aforementioned departments. The three groups were matched for age and sex. The inclusion Criteria are as follows: (1) Age ≥ 18 years; (2) Possession of a stable contact method and willingness to cooperate; (3) VM group meeting the diagnostic criteria for dVM or pVM as defined by the 2022 Bárány Society criteria, M group meeting the diagnostic criteria for migraine with aura or migraine without aura according to the International Classification of Headache Disorders, 3rd edition (ICHD-3) published by the International Headache Society (IHS) in 2018 and Hc Group should be absence of any current or past symptoms or history of headache or dizziness. Exclusion criteria applied to all groups included: (1) History of gastrointestinal diseases; (2) Use of antibiotics (including medications containing bismuth subsalicylate), acid-suppressing drugs, or microecological modulators (including probiotics/prebiotics/synbiotics) within 1 month prior to enrollment; (3) Comorbid psychiatric disorders, other chronic diseases (including cardiovascular diseases, hypertension, diabetes mellitus, malignancy, thyroid disorders, etc.), or any other major systemic illnesses; (4) Chronic use of oral medications. Both definite vestibular migraine (dVM) and probable vestibular migraine (pVM) patients meeting the diagnostic criteria were enrolled and are collectively referred to as the “VM group” throughout the manuscript.

This study was designed as an exploratory pilot investigation to generate hypotheses regarding multi-omic alterations in VM. Given the absence of prior multi-omic data in VM to inform effect size estimates, a formal *a priori* sample size calculation was not performed. We acknowledge that this sample size may limit statistical power; therefore, all findings, particularly those not surviving multiple testing correction, should be interpreted as preliminary and hypothesis-generating.

### 2.2 Data and sample collection

#### 2.2.1 Demographic and disease-related information

Demographic information [age, sex, body mass index (BMI)] and disease-related information (disease duration, attack frequency, clinical manifestations during attacks) were collected using Excel spreadsheets. Migraine patients were assessed using the Visual Analog Scale (VAS) for headache severity, and VM patients were assessed using the Dizziness Handicap Inventory (DHI) scale.

#### 2.2.2 Fecal sample collection and processing

Fecal sample collection occurred within 24 h of the collection of demographic/disease information and plasma/urine samples. Participants were instructed to collect fecal samples in the hospital according to a standardized protocol. Prior to collection, participants were asked to empty their bladder. Approximately 3 g of fecal material was collected from the middle inner part of the stool using a uniform 5 ml sterile fecal collection device. The sample was immediately labeled with a marker pen, placed on ice, sealed with parafilm within 2–4 h, and stored at −80 °C until analysis.

#### 2.2.3 Plasma sample collection and processing

Plasma sample collection occurred within 24 h of the collection of demographic/disease information and fecal/urine samples. After an overnight fast, 5 ml of peripheral blood was collected from each participant at the outpatient department or Neurology Ward IV into EDTA anticoagulant tubes. Samples were transported on ice. Plasma was separated by centrifugation at 3,000 rpm for 10 min at room temperature within 2–4 h of collection. The supernatant was aliquoted into 1.5 ml centrifuge tubes (0.2 ml per tube), labeled, sealed with parafilm, and stored at −80 °C until analysis.

#### 2.2.4 Urine sample collection and processing

Urine sample collection occurred within 24 h of the collection of demographic/disease information and fecal/plasma samples. Participants were instructed to collect mid-stream morning urine (approximately 20 ml) into sterile containers in the hospital according to a standardized protocol. Samples were transported on ice. Urine was centrifuged at 1,000 rpm for 5 min at 4 °C within 2–4 h of collection. The supernatant was filtered through a 0.22 μm membrane filter, aliquoted into 1.5 ml centrifuge tubes (1 ml per tube), labeled, sealed with parafilm, and stored at −80 °C until analysis.

### 2.3 Data processing and analysis

#### 2.3.1 Analysis of demographic and disease-related data

Statistical analyses were performed using IBM SPSS Statistics software (version 25.0). Categorical data are presented as number and percentage [*n* (%)]. Group comparisons for categorical variables were conducted using the Chi-square test or Fisher's exact test, as appropriate. Continuous data are expressed as mean ± standard deviation (SD) if normally distributed, or as median (interquartile range; IQR) if non-normally distributed. Group comparisons for continuous variables were performed using analysis of variance (ANOVA). When the assumption of homogeneity of variances was violated (assessed by Levene's test), Welch's ANOVA with Tamhane's T2 *post-hoc* test was used. A *P*-value < 0.05 was considered statistically significant.

#### 2.3.2 Fecal 16S rRNA gene sequencing and data analysis

DNA from different samples was extracted using the E.Z.N.A.^®^ Stool DNA Kit (D4015, Omega, Inc., USA) according to manufacturer's instructions. The reagent which was designed to uncover DNA from trace amounts of sample has been shown to be effective for the preparation of DNA of most bacteria. Nuclear-free water was used for blank. The total DNA was eluted in 50 μl of Elution buffer and stored at −80 °C until measurement in the PCR. The 5′ ends of the primers were tagged with specific barcods per sample and sequencing universal primers. PCR amplification was performed in a total volume of 25 μl reaction mixture containing 25 ng of template DNA, 12.5 μl PCR Premix, 2.5 μl of each primer, and PCR-grade water to adjust the volume. The PCR conditions to amplify the prokaryotic 16S fragments consisted of an initial denaturation at 98 °C for 30 s; 32 cycles of denaturation at 98 °C for 10 s, annealing at 54 °C for 30 s, and extension at 72 °C for 45 s; and then final extension at 72 °C for 10 min. The PCR products were confirmed with 2% agarose gel electrophoresis. Throughout the DNA extraction process, ultrapure water, instead of a sample solution, was used to exclude the possibility of false-positive PCR results as a negative control. The PCR products were purifyied by AMPure XT beads (Beckman Coulter Genomics, Danvers, MA, USA) and quantified by Qubit (Invitrogen, USA). The amplicon pools were prepared for sequencing and the size and quantity of the amplicon library were assessed on Agilent 2100 Bioanalyzer (Agilent, USA) and with the Library Quantification Kit for Illumina (Kapa Biosciences, Woburn, MA, USA), respectively. The libraries were sequenced on NovaSeq PE250 platform. Samples were sequenced on an Illumina NovaSeq platform according to the manufacturer's recommendations. Paired-end reads were assigned to samples based on their unique barcode and truncated by cutting off the barcode and primer sequence. Paired-end reads were merged using FLASH. Quality filtering on the raw reads was performed under specific filtering conditions to obtain the high-quality clean tags according to the fqtrim (v0.94). Chimeric sequences were filtered using Vsearch software (v2.3.4). After dereplication using DADA2, we obtained feature table and feature sequence. Alpha diversity and beta diversity were calculated by normalized to the same sequences randomly. Then according to SILVA (release 132) classifier, feature abundance was normalized using relative abundance of each sample. Alpha diversity is applied in analyzing complexity of species diversity for a sample through 5 indices, including Chao1, Observed species, Goods coverage, Shannon, Simpson, and all these indices in our samples were calculated with QIIME2. Beta diversity was calculated by QIIME2, the graphs were drawn by R package. Blast was used for sequence alignment, and the feature sequences were annotated with SILVA database for each representative sequence.

Additionally, taxonomic classification and subsequent analyses were performed using the SILVA and NT-16S databases. Based on the annotation results of Amplicon Sequence Variants (ASVs) and the ASV abundance table for each sample, species abundance tables at the kingdom, phylum, class, order, family, genus, and species levels were obtained. Compositional analysis and differential abundance analysis were conducted on the abundance tables at different taxonomic levels to identify differentially abundant taxa. This included the Kruskal–Wallis test, with *P*-values adjusted for multiple comparisons using the Benjamini–Hochberg method for False Discovery Rate (FDR) correction, applying a significance threshold of *q* < 0.05. For the LEfSe analysis, no results remained statistically significant after multiple testing correction. Therefore, in line with the exploratory nature of this study, results with an *LDA* score > 3 and an uncorrected *P* < 0.05 were considered statistically significant for exploratory purposes and are presented in a bar chart. Diagrams were implemented using the R package (v3.5.2).

#### 2.3.3 Plasma and urine metabolomics and data analysis

100 μl of sample was taken, mixed with 400 μl of extraction solution [MeOH:ACN, 1:1 (v/v)], the extraction solution contains deuterated internal standards, the mixed solution was vortexed for 30 s, sonicated for 10 min in 4 °C water bath, and incubated for 1 h at −40 °C to precipitate proteins. Then the samples were centrifuged at 12,000 rpm (RCF = 13,800 ( × g), *R* = 8.6cm) for 15 min at 4 °C. The supernatant was transferred to a fresh glass vial for analysis. The quality control (QC) sample was prepared by mixing an equal aliquot of the supernatant of samples. For polar metabolites, LC-MS/MS analyses were performed using an UHPLC system (Vanquish, Thermo Fisher Scientific) with a Waters ACQUITY UPLC BEH Amide (2.1 mm × 50 mm, 1.7 μm) coupled to Orbitrap Exploris 120 mass spectrometer (Orbitrap MS, Thermo). The mobile phase consisted of 25 mmol/L ammonium acetate and 25 ammonia hydroxide in water (pH = 9.75) (A) and acetonitrile (B). The auto-sampler temperature was 4 °C, and the injection volume was 2 μl. The Orbitrap Exploris 120 mass spectrometer was used for its ability to acquire MS/MS spectra on information-dependent acquisition (IDA) mode in the control of the acquisition software (Xcalibur, Thermo). In this mode, the acquisition software continuously evaluates the full scan MS spectrum. The ESI source conditions were set as following: sheath gas flow rate as 50 Arb, Aux gas flow rate as 15 Arb, capillary temperature 320 °C, full MS resolution as 60,000, MS/MS resolution as 15,000, collision energy: SNCE 20/30/40, spray voltage as 3.8 kV (positive) or −3.4 kV (negative), respectively.

The raw data were converted to the mzXML format using ProteoWizard and processed with an in-house program. which was developed using R and based on XCMS, for feature detection, extraction, alignment, and integration. The R package and the BiotreeDB (V3.0) were applied in metabolite identification. In this study, features were detected and metabolites were left after relative standard deviation de-noising. Then, the missing values were filled up by half of the minimum value. Also, internal standard normalization method was employed in this data analysis. The final dataset containing the information of feature number, sample name and normalized feature area was imported to SIMCA18.0.1 software package (Sartorius Stedim Data Analytics AB, Umea, Sweden) for multivariate analysis. Data was scaled and logarithmic transformed to minimize the impact of both noise and high variance of the variables. After these transformations, PCA (principal component analysis, PCA), an unsupervised analysis that reduces the dimension of the data, was carried out to visualize the distribution and the grouping of the samples. 95% confidence interval in the PCA score plot was used as the threshold to identify potential outliers in the dataset. In order to visualize group separation and find significantly changed metabolites, supervised orthogonal projections to latent structures-discriminate. analysis (OPLS-DA) was applied. Then, a 7-fold cross validation was performed to calculate the value of R2 and Q2. R2 indicates how well the variation of a variable is explained and Q2 means how well a variable could be predicted. To check the robustness and predictive ability of the OPLS-DA model, 200 times permutations was further conducted. Afterward, the R2 and Q2 intercept values were obtained. Here, the intercept value of Q2 represents the robustness of the model, the risk of overfitting and the reliability of the model, which will be the smaller the better. Furthermore, the value of variable importance in the projection (VIP) of the first principal component in OPLS-DA analysis was obtained. It summarizes the contribution of each variable to the model. Since no metabolites remained statistically significant after multiple testing correction, and in keeping with the exploratory aim of the study, metabolites with a *VIP* score > 1 and an uncorrected *P* < 0.05 were selected as meaningful for further analysis. In addition, commercial databases including KEGG (http://www.genome.jp/kegg/) was used for pathway enrichment analysis.

#### 2.3.4 Correlation analyses of differential gut genera, metabolites, and clinical features

For further exploration, correlation analyses were performed between clinical features and microbiome/metabolome results, as well as between microbiome and metabolome results themselves. Clinical measures were attack frequency [categorized as 1 (once every few months or years) or 2 (several times per month)] and DHI scores (total and subscale scores: DHI-P, DHI-F, DHI-E). Spearman correlation analysis and associated statistical graphs were performed/generated using GraphPad Prism 10.

## 3 Results

### 3.1 Demographic data

Five participants in the VM group (designated as VM1, VM8, VM9, VM12, and VM14) withdrew from the study and revoked their informed consent. Consequently, the final VM group comprised 10 patients, all female, with a mean age of 41.5 ± 14.3 years. The M group included all 15 enrolled patients (1 male, 14 females), with a mean age of 30.9 ± 8.0 years. The Hc group had a mean age of 29.1 ± 6.3 years and included 2 males and 13 females. There were no significant differences in sex distribution (Fisher's exact test, *P* = 0.772) or mean age (*P* > 0.05) among the three groups (Table 1).

**Table 1:** Age and gender differences between groups.

**Characteristic**	**VM group (*N* = 10)**	**M group (*N* = 15)**	**Hc group (*N* = 15)**	***F*/*P***	***P***
Age	41.5 ± 14.3 (31.3, 51.7)	30.9 ± 8.0 (26.5, 35.4)	29.1 ± 6.3 (25.6, 32.5)	5.74	0.007
**Gender**				0.159	0.772
Male	0 (0%)	1 (6.7%)	2 (13.3%)		
Female	10 (100%)	14 (93.3%)	13 (86.7%)		

One-way ANOVA showed a significant overall difference in age among the three groups (*P* = 0.007). However, due to unequal variances (Levene's test, *P* < 0.05), Tamhane's T2 *post-hoc* test was applied for pairwise comparisons, which revealed no significant differences between any two groups after correction for multiple comparisons: VM vs. M, *P* = 0.152; VM vs. Hc, *P* = 0.072; M vs. Hc, *P* = 0.862. Therefore, the three groups were considered comparable in terms of age distribution.

### 3.2 16S rRNA results

#### 3.2.1 ASVs analysis

The Venn diagram constructed based on ASVs (Supplementary Figure S1) illustrates compositional differences in gut microbiota among the three groups. Compared with the Hc group, the VM group shared 929 ASVs and exhibited 909 unique ASVs, while the Hc group exhibited 2,129 unique ASVs. Compared with the M group, the VM group shared 903 ASVs and exhibited 935 unique ASVs, while the M group exhibited 2,101 unique ASVs. Between the M group and the Hc group, 1,158 ASVs were shared; the M group exhibited 1,846 unique ASVs, and the Hc group exhibited 1,900 unique ASVs.

#### 3.2.2 Alpha diversity analysis

There were no significant differences in the Chao1 index, observed species, Shannon index, Simpson index, or Pielou's evenness (pielou-e) index among the three groups, suggesting no marked differences in microbial community richness or diversity. However, the Chao1 index and observed species were significantly higher in the M group compared to the VM group, indicating greater microbial community richness in the M group (Supplementary Figures S2A–E, Supplementary Table S1). Meanwhile, as shown in Supplementary Figure S2F, Goods_coverage values approached 1 in all three groups, indicating that the sequencing results adequately reflected the true microbial composition of the samples.

#### 3.2.3 Beta diversity analysis

Principal Coordinates Analysis (PCoA) scatter plots revealed noticeable differences in the clustering distribution of microbial communities among the three groups. Similarity analysis (ANOSIM) further confirmed significant separation between the groups (M vs. VM vs. Hc, *R* = 0.4175, *P* = 0.001) (Supplementary Figure S3).

#### 3.2.4 Changes in gut microbiota compositional abundance

**Figure 1:**
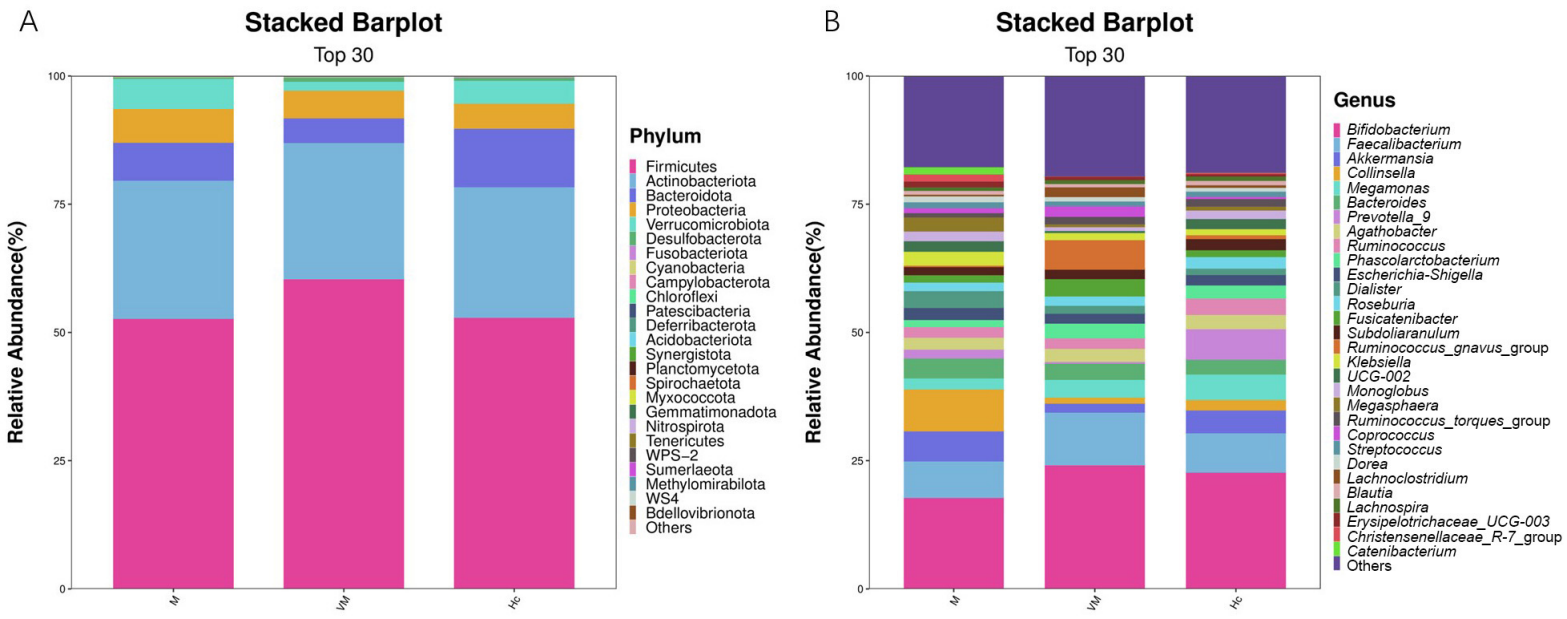
Taxonomic classification of the top 30 species in terms of abundance at phylum **(A)** and genus levels **(B)**.

We focused on compositional changes at the phylum and genus levels. Analysis of the gut microbiota composition selected the top 30 most abundant taxa, and their relative abundance distribution across groups is presented as stacked bar charts (Figure 1). At the phylum level, Firmicutes, Actinobacteriota, Bacteroidota, Proteobacteria, and Verrucomicrobiota dominated the abundance in all three groups. Within the VM group, the relative abundance of Verrucomicrobiota demonstrated a decreasing trend, while Firmicutes and others showed an increasing trend, as detailed in Figure 1A. At the genus level, compared to the other two groups, genera such as *Akkermansia* exhibited a marked decreasing trend in relative abundance, whereas the *Ruminococcus gnavus* group and others showed a marked increasing trend (Figure 1B).

#### 3.2.5 Differential abundance analysis of gut microbiota composition

Following differential abundance analysis with FDR correction, significant reductions in the abundance of Verrucomicrobiota (*q* = 0.041) and Chloroflexi (*q* = 0.022) were observed in the VM group at the phylum level compared to the other two groups. Further pairwise comparisons revealed that the relative abundance of Chloroflexi was significantly lower in the VM group compared to the M group (*q* = 0.01), while Verrucomicrobiota showed a decreasing trend approaching significance (*q* = 0.055). Neither phylum exhibited a significant difference in abundance when compared to the Hc group (Supplementary Figure S4).

At the genus level, the relative abundances of all 44 differentially abundant genera among the three groups were below 0.5%. Among these, genera including *Latilactobacillus, JG30-KF-AS9_unclassified, Parvimonas, Olsenella, Cellulomonas, Porphyromonas*, and *Peptostreptococcus* exhibited higher relative abundance in the VM group. Further pairwise comparisons within these genera revealed that compared to the Hc group, *Latilactobacillus* was significantly elevated in the VM group (*q* = 0.017); compared to the M group, *JG30-KF-AS9_unclassified* was significantly elevated (*q* = 0.021), while *Olsenella* showed an increasing trend approaching significance (*q* = 0.0502). Notably, the relative abundance of multiple genera was zero (“0”) in one or two groups, which may reflect limitations in sequencing depth or associations with factors related to the disease's pathophysiology (Supplementary Figure S5).

**Figure 2:**
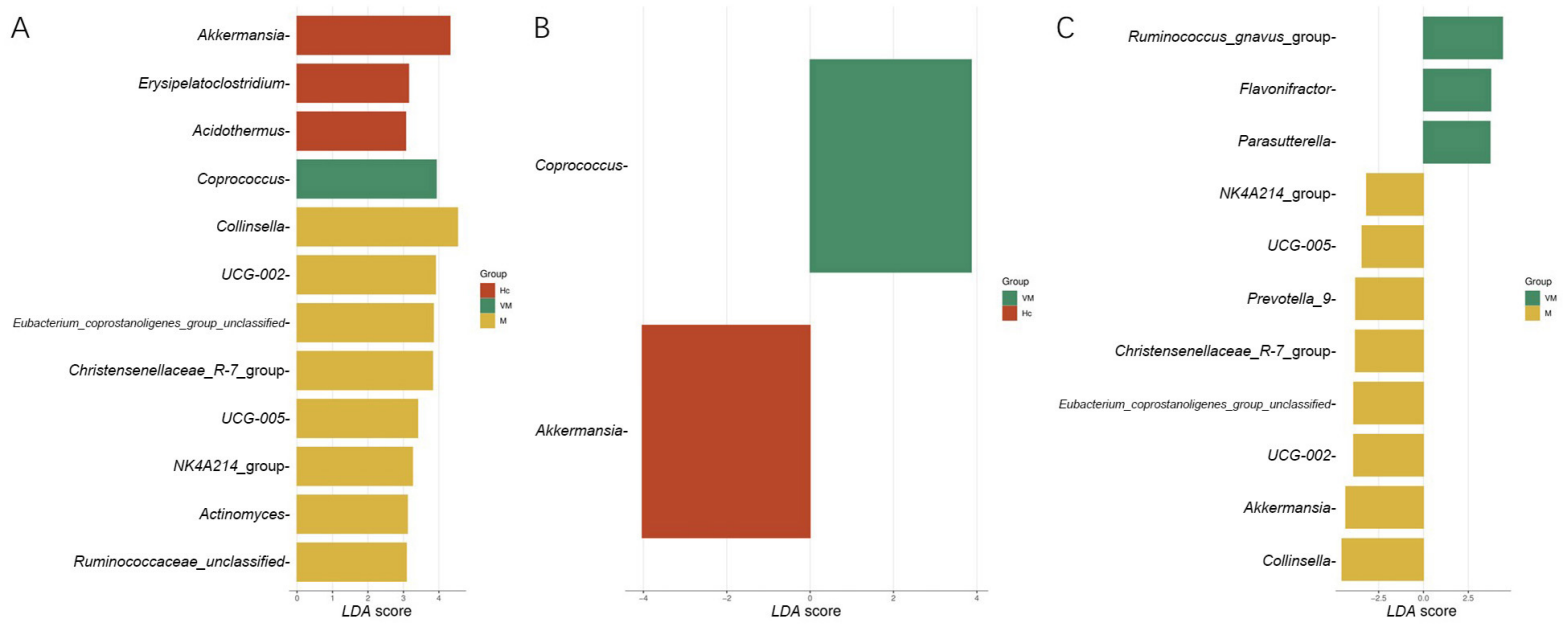
The results of LEfSe difference analysis. **(A)** The results of LEfSe difference analysis across three groups (Hc, VM, M). **(B)** The results of LEfSe difference analysis between VM and Hc group. **(C)** The results of LEfSe difference analysis between VM and M group.

Further LEfSe analysis revealed no taxa that remained statistically significant after correction for multiple comparisons. However, for exploratory purposes, we also report findings from the uncorrected analysis. At the genus level, *Coprococcus* was specifically enriched in the VM group (*LDA* = 3.91, *P* = 0.034) (Figure 2A). This enrichment was also significant when comparing the VM group to the Hc group (*LDA* = 3.87, *P* = 0.046) (Figure 2B). However, no significant enrichment of *Coprococcus* was observed in the VM group compared to the M group (*LDA* < 3, *P* > 0.05) (Figure 2C); instead, specific enrichment was found for the *Ruminococcus gnavus* group (*LDA* = 4.41, *P* = 0.020), *Flavonifractor* (*LDA* = 3.75, *P* = 0.046), and *Parasutterella* (*LDA* = 3.71, *P* = 0.027) in the VM group relative to the M group. However, no specific findings at the phylum level.

### 3.3 Plasma metabolomics results

#### 3.3.1 Raw data preprocessing results

Following preprocessing, a total of 17,311 features were retained, of which 1,157 were secondarily identified metabolites. The classification of these secondarily identified metabolites is shown in Supplementary Figure S6. Further annotation and differential analysis revealed that compared to the Hc group, the VM group exhibited 54 up-regulated and 46 down-regulated metabolites; compared to the M group, the VM group exhibited 115 up-regulated and 102 down-regulated metabolites (Supplementary Table S2).

#### 3.3.2 Orthogonal partial least squares-discriminant analysis

OPLS-DA was employed to filter out orthogonal variables unrelated to the categorical variables and separately analyze the predictive (non-orthogonal) and orthogonal components. This approach provides more reliable information regarding the group differences in metabolites and their correlation with the experimental groups. The OPLS-DA score plot demonstrated clear separation between the three sample groups and good intra-group reproducibility (Figure S7).

#### 3.3.3 Screening of differential metabolites and kegg enrichment analysis

Since no metabolites remained statistically significant after multiple testing correction among the three groups or between pairwise groups, the differential metabolites discussed below were screened based on an uncorrected *P* < 0.05 and *VIP* score > 1. It is important to note that these results carry a potential false positive risk; therefore, the subsequent analyses should be interpreted strictly as exploratory. Heatmaps were generated to visualize the top 20 differential metabolites identified in the comparisons among the three groups, between the VM and Hc groups, and between the VM and M groups (Supplementary Figure S8).

#### 3.3.4 KEGG annotation and enrichment analysis of differential metabolites

**Figure 3:**
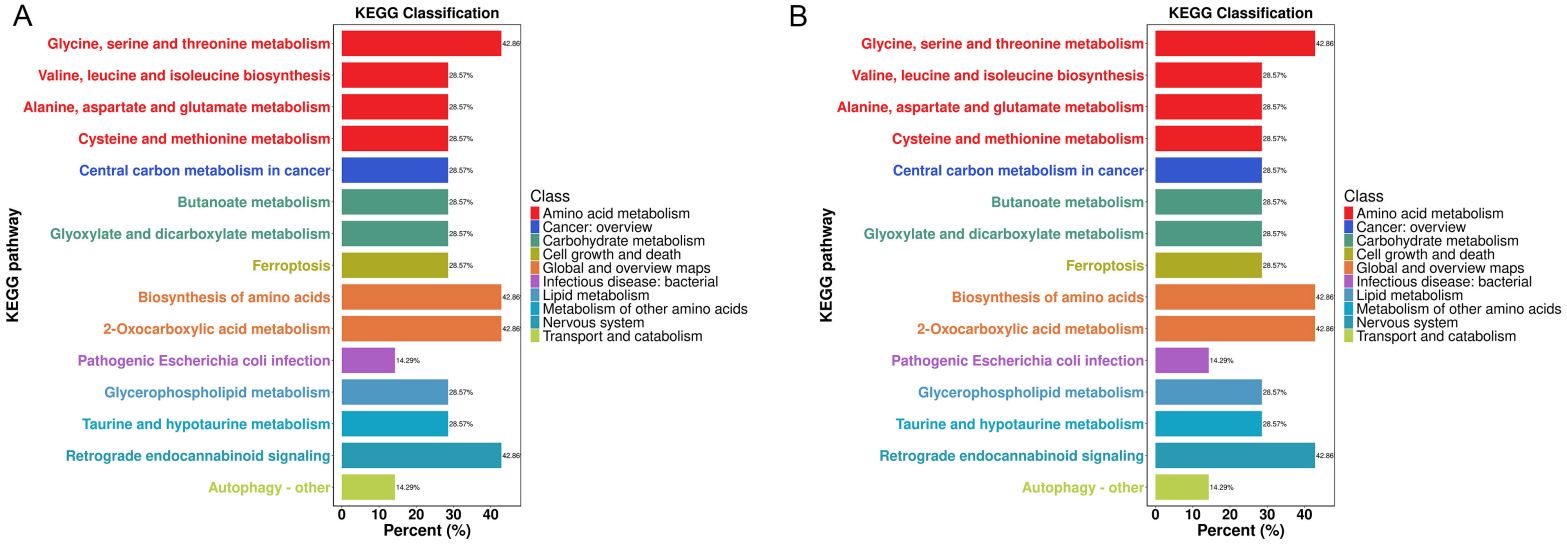
KEGG annotation analysis of differential metabolites. **(A)** KEGG annotation analysis of differential metabolites between the VM and Hc groups. **(B)** KEGG annotation analysis of differential metabolites between the VM and M groups.

KEGG annotation and enrichment analysis of all differential metabolites among the three groups revealed that the pathways with the largest proportions were 2-Oxocarboxylic acid metabolism and Biosynthesis of amino acids (Supplementary Figure S9). Among the top 20 most significant differential metabolites associated with these pathways were pyruvate and 2-ketobutyric acid. Further pairwise comparisons showed that, compared to the Hc group (Figure 3A) and the M group (Figure 3B), 2-Oxocarboxylic acid metabolism remained the most prominent biological pathway. Similarly, pyruvate and 2-ketobutyric acid were consistently among the top 20 most significant differential metabolites involved in this pathway in both comparisons (Table 2).

**Table 2:** Differential metabolites involved in the 2-Oxocarboxylic acid metabolism from KEGG annotation analysis of inter-group comparisons.

**Comparison**	**Differential metabolites**
Hc-M-VM	Pyruvate, 2-Oxobutyric acid
Hc-VM	Pyruvate, 2-Oxobutyric acid, L-glutamate
M-VM	**Pyruvate, 2-Oxobutyric acid**, 2-Aminobutyric acid, Phenylalanine

Bold entries belong to the top 20 significant differential metabolites mapped to the pathways in specific inter-group comparison.

#### 3.3.5 KEGG topological analysis

**Figure 4:**
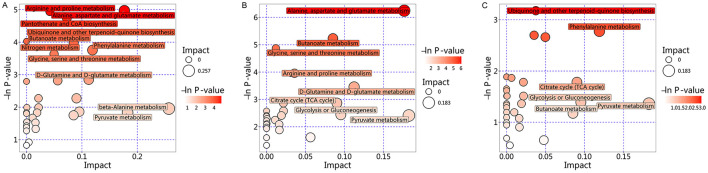
KEGG topological analysis of differential metabolites. **(A)** KEGG topological analysis of differential metabolites among the three groups. **(B)** KEGG Topological analysis of differential metabolites between the VM and Hc groups. **(C)** KEGG topological analysis of differential metabolites between the VM and M groups. The x-axis (Impact) represents the topological influence factor, with circle size proportional to pathway enrichment level. The y-axis (-ln *P*) displays the negative natural logarithm of *P*-values (base e). Color intensity denotes statistical significance, where -ln *P* > 3 (equivalent to *P* < 0.05) indicate significance thresholds, with darker hues reflecting higher confidence.

Further KEGG topological analysis of differential metabolites among the three groups (Figure 4A) indicated that the Alanine, aspartate and glutamate metabolism pathway was significantly enriched (Rich factor = 0.177, *P* = 0.007). Pyruvate was among the top 20 most significant differential metabolites associated with this pathway (Supplementary Table S4). Pairwise comparisons revealed that analysis of differential metabolites between the VM and Hc groups (Figure 4B) also showed significant enrichment of the Alanine, aspartate and glutamate metabolism pathway (Rich factor = 0.177, *P* = 0.002), with pyruvate again being among the associated top 20 most significant metabolites (Supplementary Table S4). However, when compared to the M group (Figure 4C), this pathway was not significantly enriched (Rich factor = 0, *P* = 0.199). Instead, only the Ubiquinone and other terpenoid–quinone biosynthesis pathway showed a statistically significant difference (Rich factor = 0.038, *P* = 0.042), although its enrichment level was not prominent. Furthermore, this latter pathway did not include any of the top 20 most significant differential metabolites (Supplementary Table S4).

### 3.4 Urine metabolomics results

#### 3.4.1 Raw data preprocessing results

Following preprocessing, a total of 26,044 features were retained, of which 2,608 were secondarily identified metabolites. The classification of these secondarily identified metabolites is shown in Supplementary Figure S10. Further annotation and differential analysis revealed that compared to the Hc group, the VM group exhibited 11 up-regulated and 40 down-regulated metabolites; compared to the M group, the VM group exhibited 4 up-regulated and 161 down-regulated metabolites (Supplementary Table S3).

#### 3.4.2 Orthogonal partial least squares-discriminant analysis

OPLS-DA was employed to filter out orthogonal variables unrelated to the categorical variables and separately analyze the predictive (non-orthogonal) and orthogonal components. This approach provides more reliable information regarding the group differences in metabolites and their correlation with the experimental groups. The OPLS-DA score plot demonstrated clear separation between the three sample groups and good intra-group reproducibility (Supplementary Figure S11).

#### 3.4.3 Screening of differential metabolites and kegg enrichment analysis

Same as the plasma results, since no metabolites remained statistically significant after multiple testing correction among the three groups or between pairwise groups, the differential metabolites discussed below were screened based on an uncorrected *P* < 0.05 and *VIP* score > 1. It is important to note that these results carry a potential false positive risk; therefore, the subsequent analyses should be interpreted strictly as exploratory. Heatmaps were generated to visualize the top 20 differential metabolites identified in the comparisons among the three groups, between the VM and Hc groups, and between the VM and M groups (Supplementary Figure S12).

#### 3.4.4 KEGG annotation and enrichment analysis of differential metabolites

KEGG annotation and enrichment analysis of all differential metabolites among the three groups identified Tyrosine metabolism and ABC transporters as the pathways with the largest proportions (Supplementary Figure S13). Among the top 20 most significant differential metabolites associated with these pathways, Norepinephrine featured prominently.

**Figure 5:**
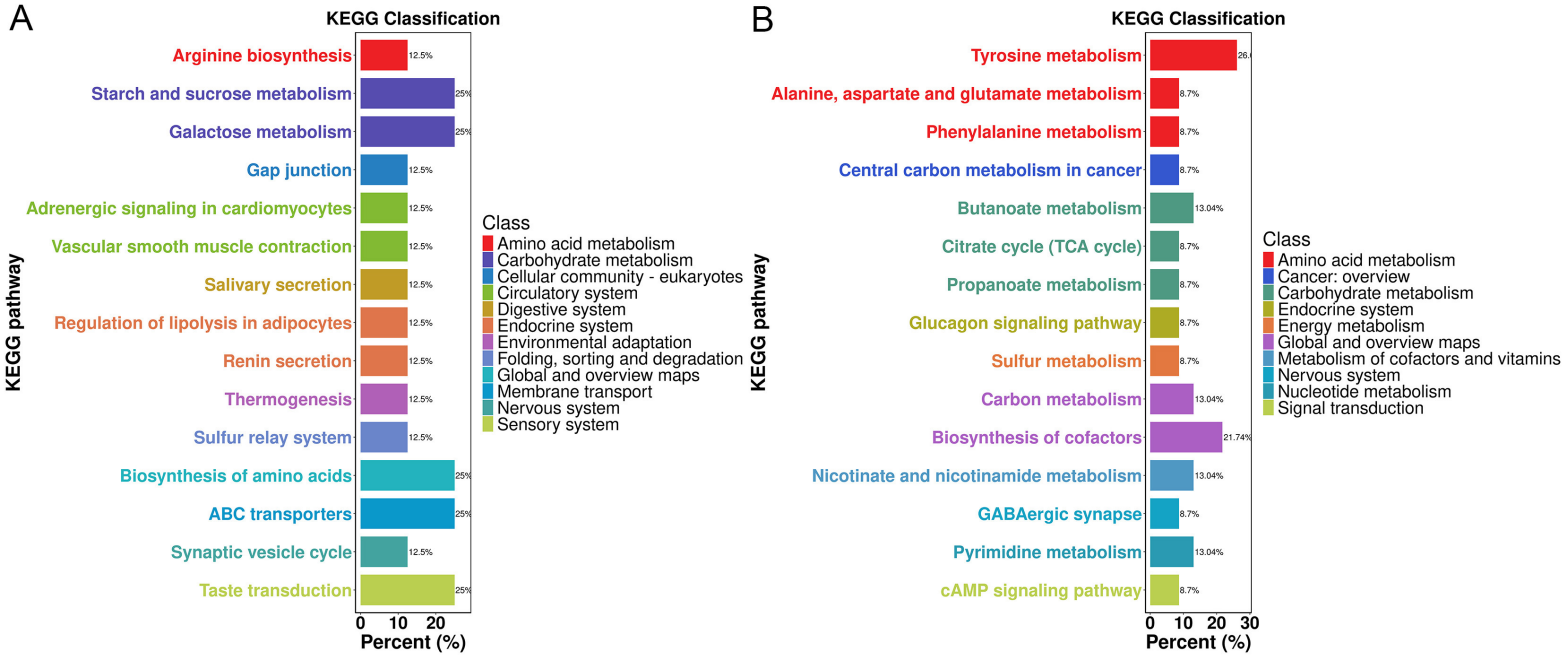
KEGG annotation analysis of differential metabolites. **(A)** KEGG annotation analysis of differential metabolites between the VM and Hc groups. **(B)** KEGG annotation analysis of differential metabolites between the VM and M groups.

Further pairwise comparisons revealed that, compared to the Hc group (Figure 5A), the pathways with the largest proportions were Starch and sucrose metabolism, Galactose metabolism, Biosynthesis of amino acids, ABC transporters, and Taste transduction. Norepinephrine (NE) was among the top 20 most significant differential metabolites associated with these pathways. In contrast, when compared to the M group (Figure 5B), Tyrosine metabolism and Biosynthesis of cofactors were the most prominent pathways; however, none of their associated differential metabolites ranked within the top 20 most significant.

#### 3.4.5 KEGG topological analysis

**Figure 6:**
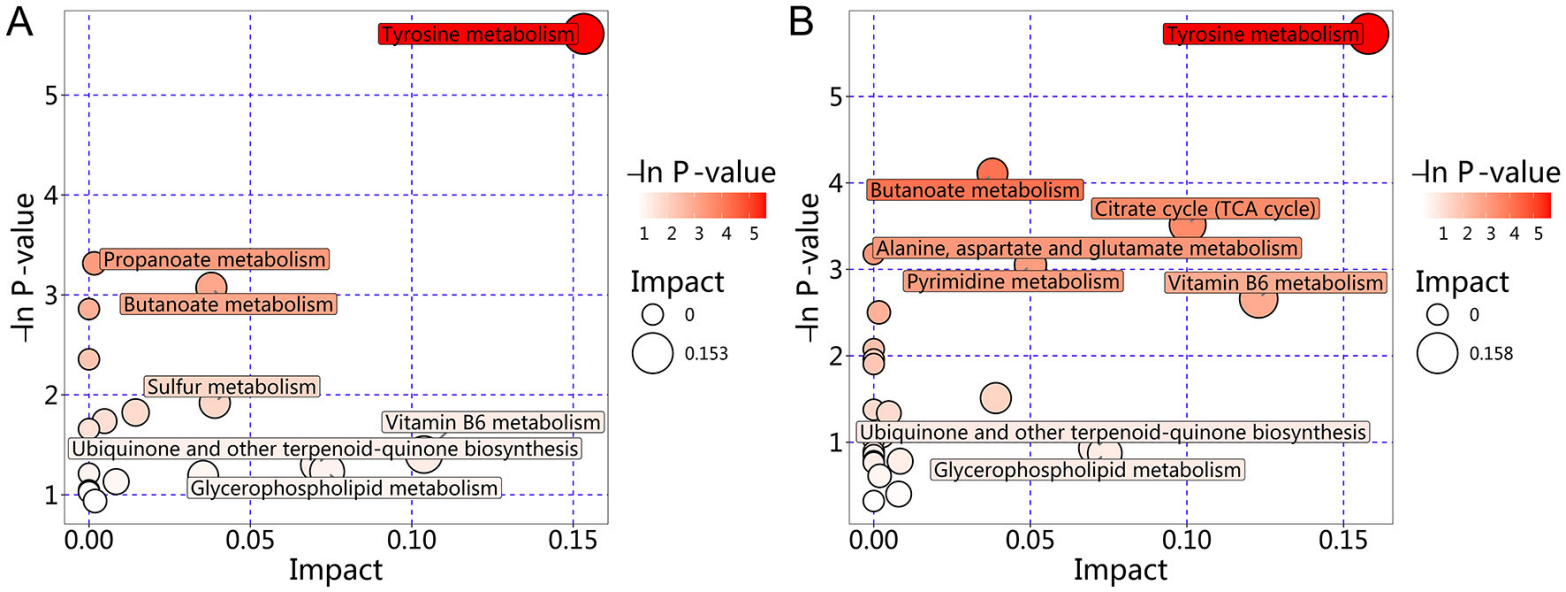
KEGG topological analysis of differential metabolites. **(A)** KEGG Topological Analysis of Differential Metabolites among the three groups. **(B)** KEGG Topological Analysis of Differential Metabolites between the VM and M groups. The x-axis (Impact) represents the topological influence factor, with circle size proportional to pathway enrichment level. The y-axis (-ln *P*) displays the negative natural logarithm of *P*-values (base e). Color intensity denotes statistical significance, where -ln *P* > 3 (equivalent to *P* < 0.05) indicate significance thresholds, with darker hues reflecting higher confidence.

Further KEGG topological analysis of differential metabolites among the three groups (Figure 6A) indicated significant enrichment of the Tyrosine metabolism pathway (Rich factor = 0.153, *P* = 0.004), with NE featuring among the top 20 most significant associated metabolites. Analysis of differential metabolites between the VM and Hc groups yielded no statistically significantly enriched pathways. However, comparison with the M group (Figure 6B) showed higher enrichment levels for the Tyrosine metabolism pathway (Rich factor = 0.158, *P* = 0.003) and the Citrate cycle (TCA cycle) pathway (Rich factor = 0.100, *P* = 0.030). Neither pathway included any of the top 20 most significant differential metabolites.

### 3.5 Correlation analyses of differential gut genera, metabolites, and clinical features

#### 3.5.1 Correlation between differential gut genera and clinical features

To further explore the gut microbiota-disease link, we analyzed correlations between the relative abundance of specific genera and clinical measures in the VM group. The genera included were those with high relative abundance and significant intergroup differences (*Latilactobacillus, JG30-KF-AS9_unclassified, Parvimonas, Olsenella, Cellulomonas, Porphyromonas, Peptostreptococcus*) and *Coprococcus*, which was specifically enriched in the VM group per LEfSe analysis. *Parvimonas* correlated positively with attack frequency (*r* = 0.7307, *P* = 0.0250). *JG30-KF-AS9_unclassified* (*r* = 0.8845, *P* = 0.0011) and *Olsenella* (*r* = 0.6838, *P* = 0.0330) correlated positively with DHI-P scores. *Peptostreptococcus* correlated positively with DHI-F scores (*r* = 0.6831, *P* = 0.0350). *JG30-KF-AS9_unclassified* (*r* = 0.7964, *P* = 0.0084) and *Porphyromonas* (*r* = 0.7771, *P* = 0.0120) correlated positively with total DHI scores. No significant correlations were found between any genera and DHI-E scores (Supplementary Figure S14A–D).

#### 3.5.2 Correlation between differential metabolites and clinical features

We next analyzed correlations between plasma pyruvate, urinary NE, and clinical features (attack frequency, DHI scores) in the VM group. Plasma pyruvate showed a positive correlation with DHI-P scores (*r* = 0.7152, *P* = 0.0225; Supplementary Figure S15) but not with other clinical measures. Urinary NE showed no significant correlations with any clinical features analyzed.

#### 3.5.3 Correlation between differential metabolites and differential gut genera

Correlation analyses were performed between the relative abundance of the differential genera and the levels of plasma pyruvate and urinary NE. Plasma pyruvate correlated positively with the relative abundance of *Cellulomonas* (*r* = 0.6727, *P* = 0.0390; Supplementary Figure S16). No other significant correlations were observed between the metabolites and the other genera. Urinary NE showed no significant correlations with any of the differential genera.

## 4 Discussion

Considering that the metabolomics and LEfSe results were not corrected for multiple testing, the following discussion involving the differential metabolites and specific microbial taxa identified (e.g., *Coprococcus*) must be interpreted as exploratory and hypothesis-generating, and is grounded in previous literature and reasonable inference.

### 4.1 Plasma metabolomics: pyruvate and energy metabolism

Plasma metabolomics revealed pyruvate as a key differential metabolite involved in several enriched pathways. Its average level was significantly higher in VM patients compared to the other two groups. At a deeper exploratory level of correlations with clinical features, plasma pyruvate showed a positive correlation with DHI-P subscale scores, which primarily reflects the disease's impact on physical activity limitations. As a crucial molecule in energy metabolism, pyruvate accumulation appears indicative of an impaired energy generation state.

The observed elevation could thus be interpreted as a potential marker of altered energy metabolism in VM. This observation is consistent with existing migraine metabolic theories, which propose migraine as a response to cerebral energy deficiency or oxidative stress exceeding antioxidant capacity, with migraine attacks potentially serving to restore brain energy homeostasis and reduce detrimental oxidative stress levels (19). Functional imaging studies suggest impaired mitochondrial oxidative phosphorylation in the brains of migraine patients across different phases (20). Specific pathophysiological mechanisms related to metabolic factors involve hypothalamic activation during the early stages of an attack, triggered by sensing metabolic changes in the brain, which subsequently influences hypothalamic-brainstem neural networks to promote migraine initiation (21). Cortical spreading depression (CSD) susceptibility is also modulated by metabolic factors. Inhibition of brain glycogen lowers the CSD threshold (22), and pharmacological inhibition of mitochondria before hypoxia significantly promotes CSD in rat hippocampal slices (23). Furthermore, CSD induces oxidative stress within the trigeminal nerve system (TNS) (24), activating TRPA1 and ASIC channels, which further promotes the release of calcitonin gene-related peptide (CGRP) from meningeal nociceptors (25). Metabolic changes have also been found to directly alter the activity of central trigeminovascular nociceptors (26).

It should be noted, however, that the evidence cited above is indirect in relation to migraine, not VM research. Our pyruvate-VM association is preliminary and does not establish causality. Nonetheless, given the substantial pathophysiological overlap thought to exist between migraine and vestibular pathways, this finding provides a rationale for future investigation. Thus, subsequent research could integrate neuroimaging techniques to elucidate relevant brain network structures, or utilize animal models to understand the relationship between migraine-associated vestibular pathways across brain regions and energy imbalance adaptation.

### 4.2 Gut microbiota: genus-level alterations and clinical correlations

In the microbiome analysis, we noted that the observed statistical differences were more pronounced at the genus level. Thus, we have chosen to focus our discussion primarily on the genus-level findings. Moreover, microbial functions, such as metabolite production, immune regulation and barrier maintenance are often conserved and specific at the genus level. This makes genus-level results more biologically interpretable and functionally relevant, which may also explain why the differences were more distinct at this level. Furthermore, from a translational perspective, genus-level signatures offer more precise targets for future mechanistic research or potential microbiome-based interventions, such as probiotics, prebiotics, or phage therapy.

In this study, at the genus level, FDR-corrected Kruskal-Wallis analysis identified significantly higher abundance in the VM group for several genera: *Latilactobacillus, JG30-KF-AS9_unclassified, Parvimonas, Olsenella, Cellulomonas, Porphyromonas*, and *Peptostreptococcus*. Subsequent pairwise comparisons confirmed significantly higher *Latilactobacillus* vs. Hc and significantly higher *JG30-KF-AS9_unclassified* vs. M, with *Olsenella* showing an increasing trend. Further correlation analyses revealed specific associations: the relative abundance of *Parvimonas* correlated positively with attack frequency; *JG30-KF-AS9_unclassified* and *Olsenella* showed positive correlations with scores of the DHI-P subscale (reflecting the physical domain); *Peptostreptococcus* correlated positively with scores of the DHI-F subscale (reflecting the functional domain), and both *JG30-KF-AS9_unclassified* and *Porphyromonas* were positively correlated with the total DHI score. Naydenova et al. (27) reported increased abundance of several Gram-negative bacilli within the genus *Porphyromonas* in the saliva and oropharyngeal microbiota of migraine patients. While gut microbiota studies specifically linking these genera to migraine are limited. To further investigate the relationship between these genera and pyruvate-related metabolism, correlation analysis revealed a positive association between plasma pyruvate and the relative abundance of *Cellulomonas*.

We subsequently reviewed the relevant literature, for *Parvimonas, Porphyromonas*, and *Peptostreptococcus*, human disease associations exist. The link between *Parvimonas* and pyruvate remains unclear, although its increased abundance in oral squamous cell carcinoma patients correlated with predicted enrichment of pyruvate metabolism pathways in metagenomic functional analysis, a pattern also noted for *Peptostreptococcus* in the same study (28). Furthermore, research on *Peptostreptococcus* suggests its capacity to consume pyruvate (29). *Porphyromonas* gingivalis, a key periodontal pathogen within this genus, also consumes pyruvate and was shown in a mouse periodontitis model to promote glycolysis by increasing pyruvate kinase 2 expression in osteoclasts (30).

Relationships between pyruvate and *Latilactobacillus, JG30-KF-AS9_unclassified, Olsenella*, or *Cellulomonas* remain largely unexplored, with existing studies often focusing on specific strains exhibiting diverse functions (31, 32). We also noted *Coprococcus* in relation to pyruvate. An observational study found higher blood pyruvate levels alongside lower *Coprococcus* abundance in ulcerative colitis patients vs. controls (33). Wen et al. demonstrated that Gastrodia-Uncaria might alleviate nitroglycerin-induced hyperalgesia in rats by modulating gut microbiota, including increasing beneficial genera like *Coprococcus*_1, thereby influencing amino acid metabolism (e.g., L-tryptophan) (34). Similar to the inconsistency observed with *Porphyromonas*, although the directionality of *Coprococcus*-pyruvate association in our study may differ from some reports, *Coprococcus* remains a genus of interest due to its beneficial roles in neuropsychiatric research [e.g., butyrate production (35)], correlating with higher quality-of-life metrics in large cohorts (35). We tentatively propose that the observed patterns, including pyruvate trends, might reflect adaptive compensatory processes. However, influences from sample size limitations, variations in disease pathology stages among subjects, significance thresholds, and potential confounding factors necessitate further validation.

### 4.3 Urine metabolomics: tyrosine pathway and norepinephrine

Bioinformatic analysis of urine metabolomics highlighted the tyrosine metabolism pathway as a common feature while NE was identified as a key metabolite within this pathway. While the urine metabolomics results, including those for NE, did not survive multiple testing correction, the observed pattern within the tyrosine metabolism pathway remains intriguing for hypothesis generation. It is critical to emphasize that the following discussion is speculative and based primarily on evidence from migraine studies, not VM.

Tyrosine is an amino acid precursor for catecholamines, including dopamine (DA) and NE by hydroxylase, as well as trace amines such as tyramine (TYR), octopamine (OCT), and synephrine (SYN) by decarboxylase (36). D'Andrea et al. (37) have conducted extensive related research on migraine. Initially, they found elevated plasma levels of OCT and SYN in migraine patients compared to controls (38). Subsequent clinical studies revealed that plasma DA, NE and TYR levels were higher in Chronic Migraine (CM) patients, while plasma TYR levels were correlated positively with the duration of CM (37). They believed that a metabolic shift in tyrosine metabolism might occur in migraine patients. Given the differing energy requirements of tyrosine hydroxylase (high demand for efficient energy supply) vs. tyrosine decarboxylase (low demand), and postulating impaired mitochondrial function in migraine, tyrosine might favor the less energy-demanding decarboxylase pathway. This shift would lead to increased synthesis of trace amines like TYR and OCT, alongside decreased NE production. Furthermore, akin to a “use it or lose it” principle, the efficiency of mitochondrial function might progressively decline over time with increasing attack frequency. The observed positive correlation between circulating TYR levels and CM duration aligns with this hypothesis (37). Furthermore, trace amine-associated receptors are located in the olfactory bulb, limbic system, amygdala, hypothalamus, extrapyramidal system, and locus coeruleus (LC) regions constituting key components of the pain matrix involved in modulating pain thresholds (39). Neuronal function within the pain matrix is primarily modulated by synapses utilizing DA and NE as neurotransmitters, while OCT acts as a neuromodulator. The observed abnormalities in circulating may suggest a non-physiological imbalance between neurotransmitters and neuromodulators, which may be linked to hypothalamic dysfunction in migraine patients, abnormalities in the sympathetic nervous system (like vertigo) and dysfunction in autonomic nervous system nuclei. Such dysregulation may subsequently activate the trigeminal nucleus, leading to the release of CGRP into the cerebral circulation and ultimately contributing to headache initiation (40).

In this study, the aforementioned trace amines were not detected in plasma. TYR was detected in urine, but no significant differences were observed among groups (see Supplementary Figure S1). This lack of difference may be attributed to limited sample size and substantial dietary variations. Although we performed correlation analyses between urinary NE and clinical features, no significant associations were found. Furthermore, we compared NE levels in plasma and urine (see Supplementary Figures S2A, B), while no significant differences emerged, the relative levels followed the pattern VM > M > Hc. Conversely, urinary NE excretion followed VM < M < Hc, with a significant difference observed between the VM and Hc groups. This discrepancy requires cautious interpretation. First, NE did not survive multiple testing correction in our study. Second, plasma and urine NE levels are influenced by distinct physiological processes, including differential renal clearance, hydration status, excretion dynamics, and circadian rhythms, which may confound direct comparisons and obscure underlying group differences.

Therefore, while our NE findings do not directly align with those in migraine patients reported by D' Andrea et al. (37), both sets of results appear to suggest an increased trend in circulating NE utilization in migraine patients which may reflect underlying impairments in energy metabolism. Regarding energy metabolism dysfunction in migraine, Petit et al. (41) proposed a link between abnormal brain glycogen metabolism and symptoms like headache, with NE potentially acting as a key neurotransmitter. External factors like stress promote wakefulness by enhancing glutamatergic transmission in the brainstem and firing of noradrenergic LC neurons, elevating NE levels in LC projection areas, further promoting glycogenolysis and escalating energy demands. Stress is a recognized trigger for both VM and M (42), serotonin-norepinephrine reuptake inhibitors, used preventatively for both conditions, effectively alleviate symptoms in some cases (43). Consequently, hypotheses regarding energy metabolism dysfunction and neurotransmitter-neuromodulator imbalance in VM/M patients remain compelling but hold significant implications, pending validation in larger, rigorously designed cohorts.

### 4.4 Limitations and future directions

The exploratory findings presented above should be interpreted in light of several limitations, many of which have been touched upon in the preceding discussions and are now summarized together. First, as discussed in the plasma metabolomics section, the metabolomics findings did not survive multiple testing correction and should therefore be interpreted as exploratory. Second, the relatively small sample size limits statistical power and increases the risk of both false negatives and false positives. This may have contributed to the lack of significant findings after multiple testing correction in the metabolomics data, and constrains the generalizability of our results to the broader VM population. Third, the cross-sectional design precludes causal inferences, a point relevant to the correlational analyses throughout this study. Fourth, as noted in the urine metabolomics discussion, the interpretation of discordant plasma and urinary NE patterns is complicated by physiological confounders such as renal clearance and circadian variation. Fifth, the use of 16S rRNA sequencing limits functional inference at the species level, as acknowledged in the microbiota discussion. Additionally, unmeasured factors including dietary intake and lifestyle variations may have influenced the results despite efforts to control for confounders. These limitations should be considered when evaluating the conclusions of this study.

Despite these limitations, this first multi-omics study in VM provides novel insights into the pathophysiology and potential therapeutic strategies, particularly concerning the interplay between gut microbiota and energy metabolism regulation. Future research should focus on larger-scale clinical studies and experimental validation of our findings. This includes more comprehensive analyses accounting for disease subtypes and confounding factors, as well as *in vitro* or *in vivo* experiments to test metabolite-related hypotheses. To overcome 16S rRNA gene sequencing depth limitations, future studies could employ whole-genome sequencing (WGS). Integrating WGS with fecal metabolomics and other multi-omics approaches would provide more accurate and direct evidence for establishing causal links. Additionally, inspired by the “seesaw theory” (44), longitudinal cohort studies could dynamically monitor the impact of factors such as pharmacological interventions and environmental exposures on gut microbiota structure. Identifying stable vs. dynamic microbial modules within this framework holds promise for informing personalized treatment approaches.

## 5 Conclusions

In this first multi-omics study of vestibular migraine, we observed distinct gut microbiota composition in VM patients compared to both migraine patients and healthy controls, particularly at the genus level. Plasma metabolomics revealed alterations involving pyruvate and amino acid metabolism pathways, suggesting potential energy metabolism disturbances in VM. Urine metabolomics highlighted the tyrosine metabolism pathway, with norepinephrine emerging as a key metabolite of interest. Collectively, these findings provide preliminary evidence for the involvement of gut dysbiosis, metabolic perturbations, and neurotransmitter-related pathways in the pathophysiology of VM. However, given the exploratory nature of this study and the limitations discussed above—particularly the uncorrected metabolomics findings and modest sample size—these results should be considered hypothesis-generating rather than confirmatory. Future studies with larger cohorts, rigorous multiple testing correction, and functional validation are warranted to elucidate the causal mechanisms underlying these observed associations and to explore their therapeutic implications.

## Data Availability

The raw data supporting the conclusions of this article will be made available by the authors, without undue reservation.
