# Supplementary material for: Gut microbiota 16S rRNA profiling with plasma and urine metabolomics in vestibular migraine
Source: Front Neurol. 2026 Mar 19;17:1722220. doi: 10.3389/fneur.2026.1722220 (PMC13043340; doi:10.3389/fneur.2026.1722220)
Supplement: Supplementary file 1 [file Data_Sheet_1.docx]

# Supplementary


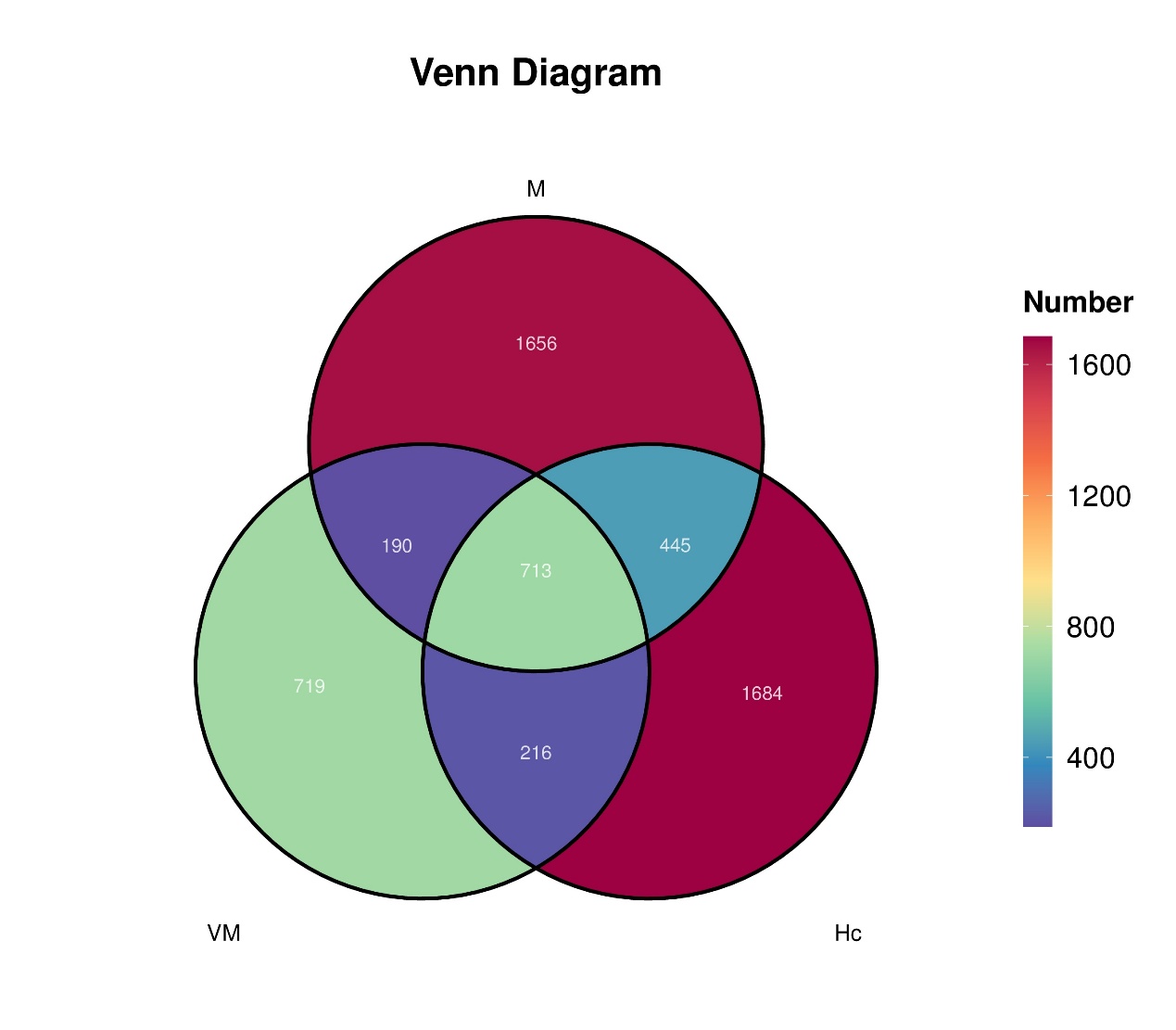


**Figure S1. The Venn diagram of ASVs in three groups**

Note. Color coding denotes experimental groups, with color intensity reflecting quantitative measures: darker hues indicate higher values.


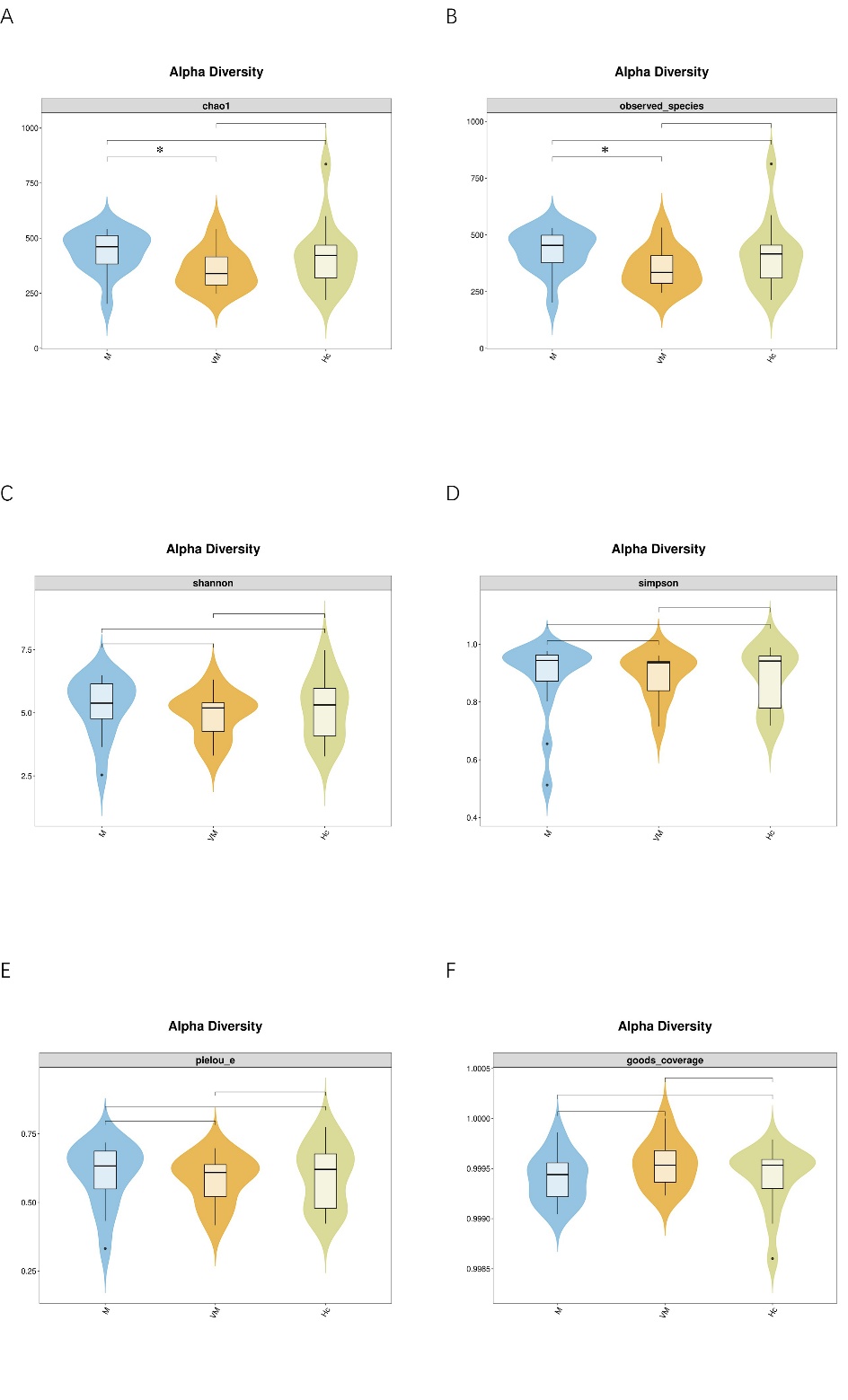


**Figure S2. The analysis of alpha diversity in three groups.**

A-F shows the results of Chao1, observed_ species, Shannon, Simpson, pielou-e and Goods _coverage. (**P<*0.05, ***P<*0.01).


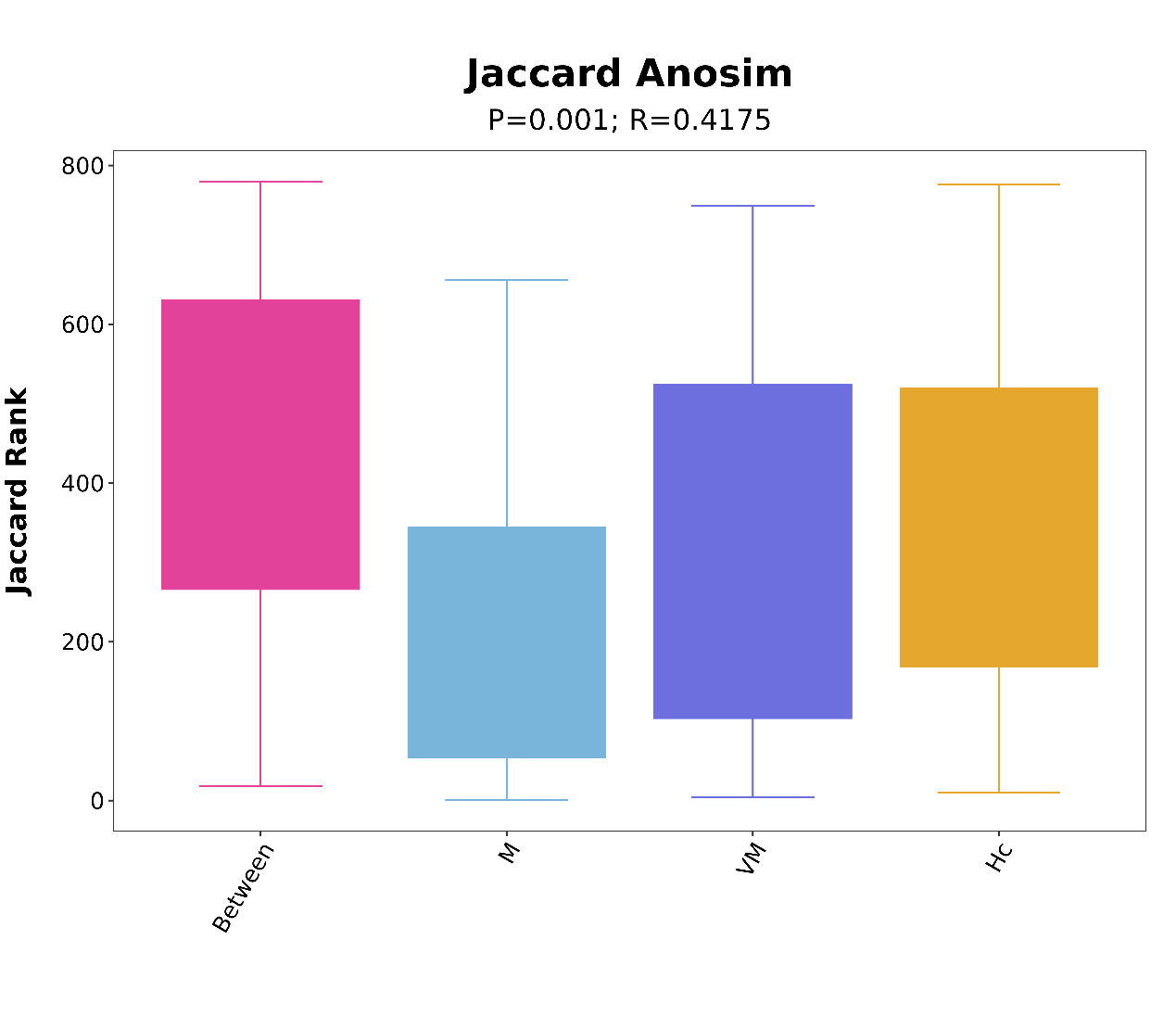


**Figure S3. The analysis of β diversity in three groups.**

A. Scatter plot of Beta diversity analysis for three groups of samples based on PCoA.

B. Box plot of Anosim similarity analysis for three groups of samples (R=0.4175，*P*=0.001).


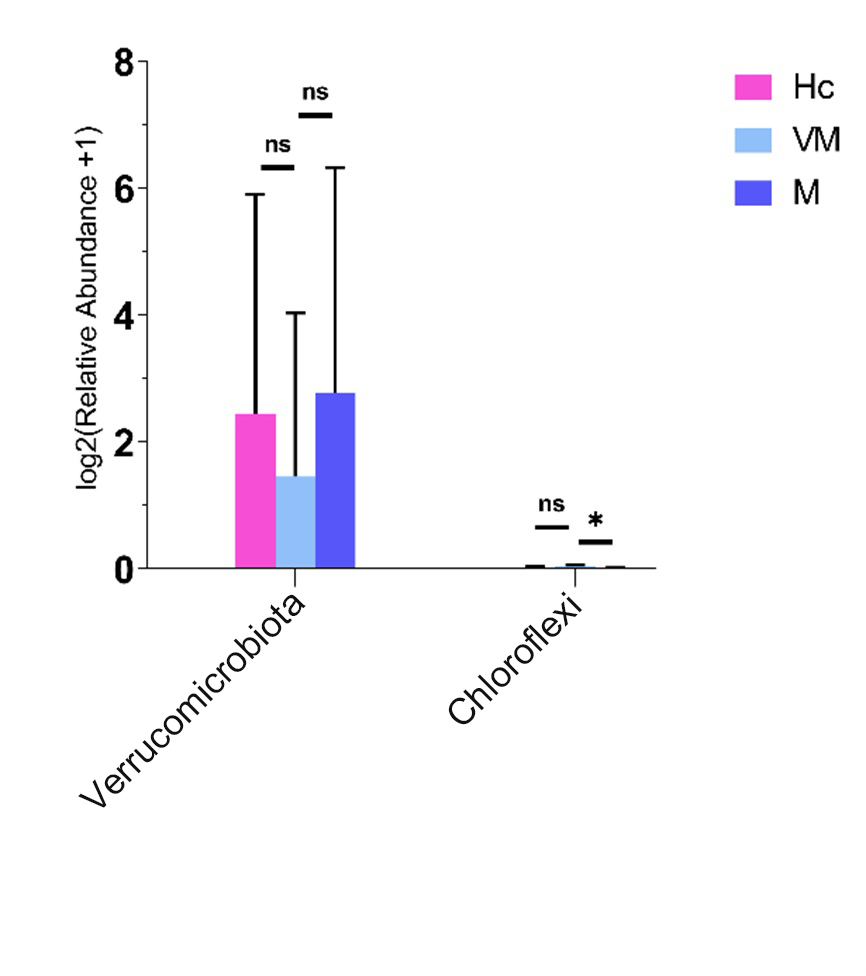


**Figure S4. Phylum-level gut microbiota composition differences among groups**

Note. “ns”: not significant, “*”: *q*<0.05


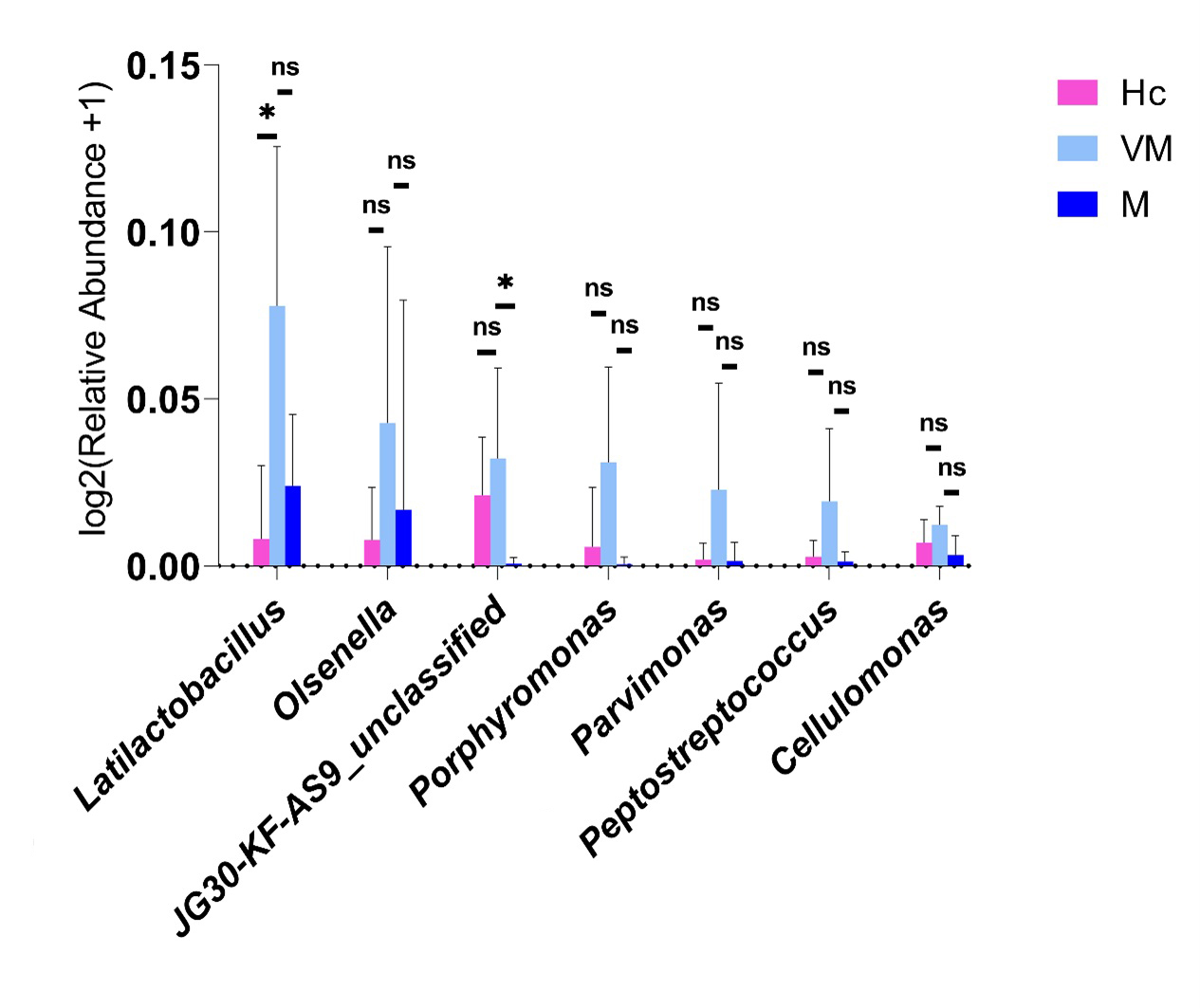


**Figure S5. Genus-level gut microbiota composition differences among groups**

Note. “ns”: not significant, “*”: *q*<0.05


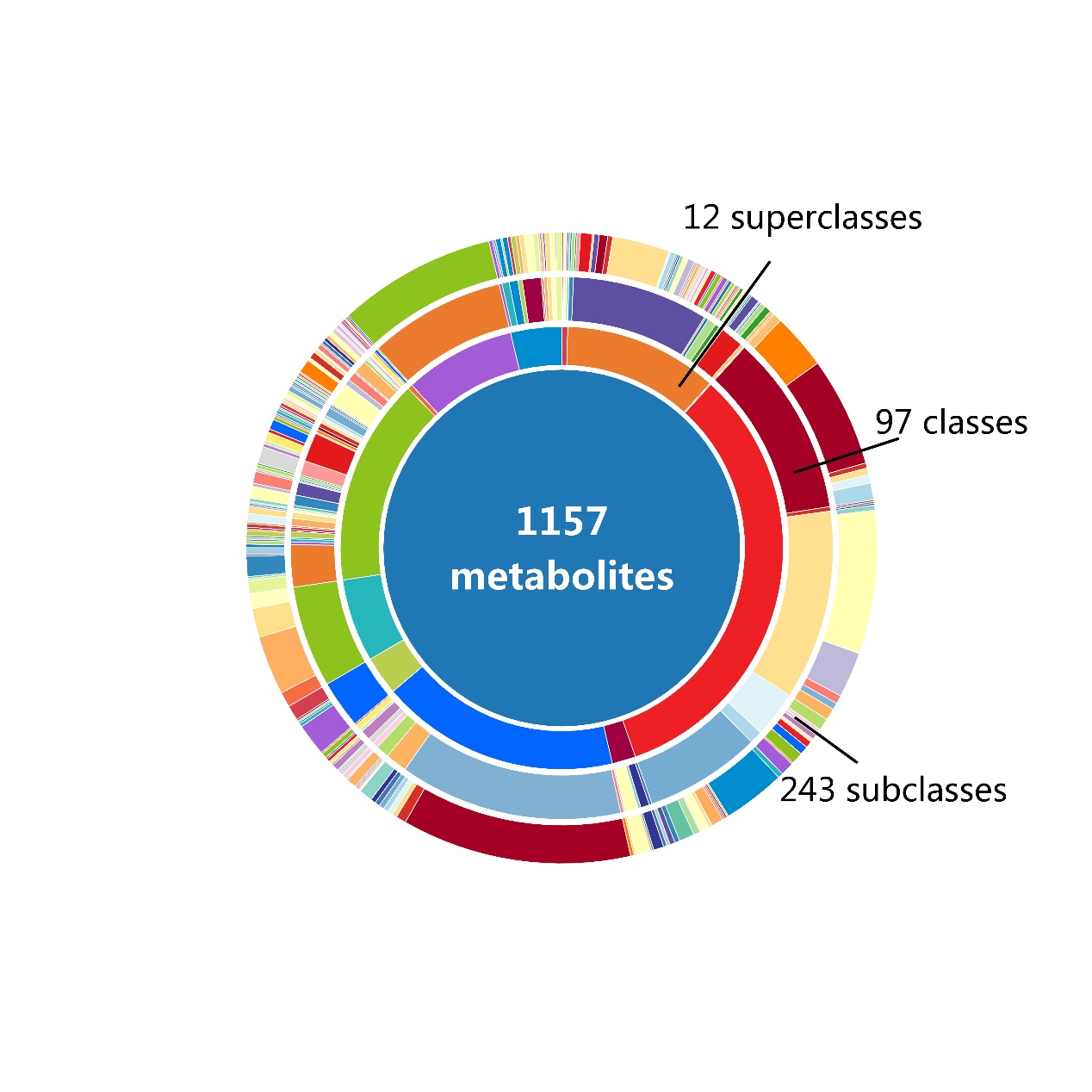


## Figure S6. The circular chart of metabolite classification.


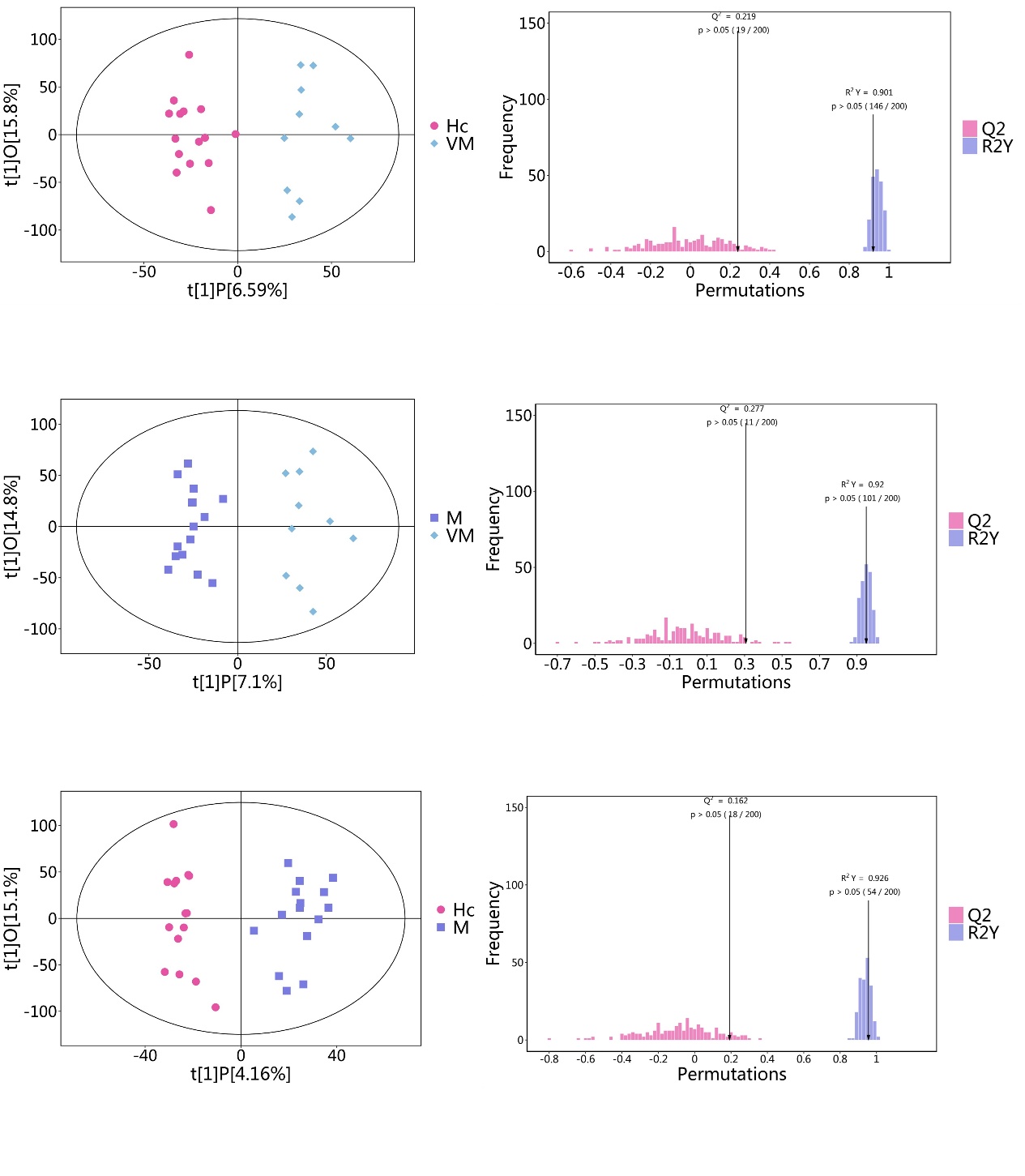


**Figure S7. Scatter plots of OPLS - DA model scores for pairwise comparisons among the three groups (left) and histogram of permutation test results of the OPLS - DA model (right).**

The red bars represent the number of occurrences of Q2 values obtained from the permutation test, and the blue bars represent the number of occurrences of R2Y values obtained from the permutation test. The p value = the number of random models in the permutation test that are superior to the original model / the total number of random models in the permutation test. Generally, when P<0.05, the model is considered optimal.


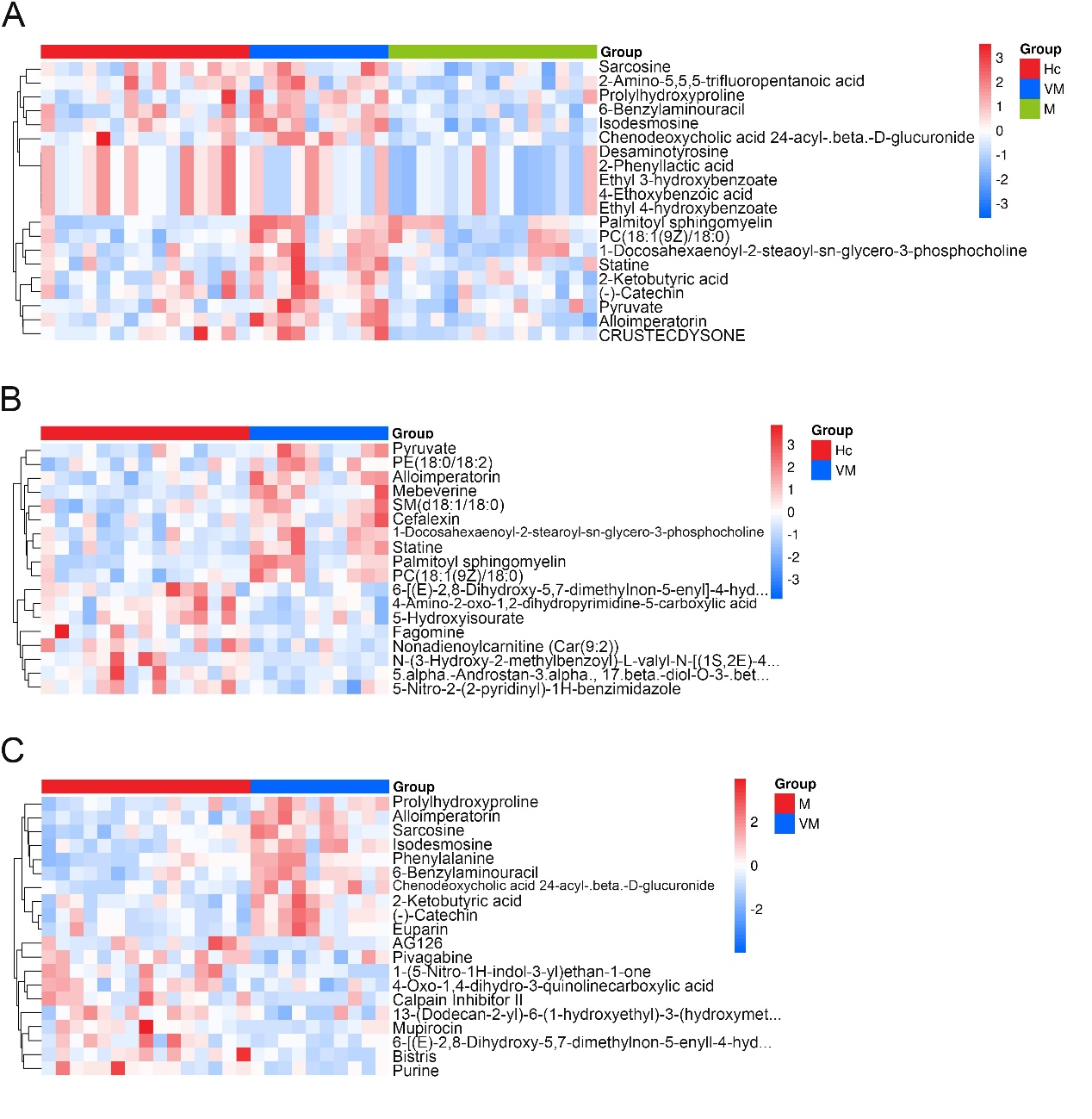


**Figure S8. The top 20 metabolites with significant differences.**

A. The top 20 metabolites with significant differences among the three groups.

B. The top 20 metabolites with significant differences between the VM and Hc groups.

C. The top 20 metabolites with significant differences between the VM and M groups.


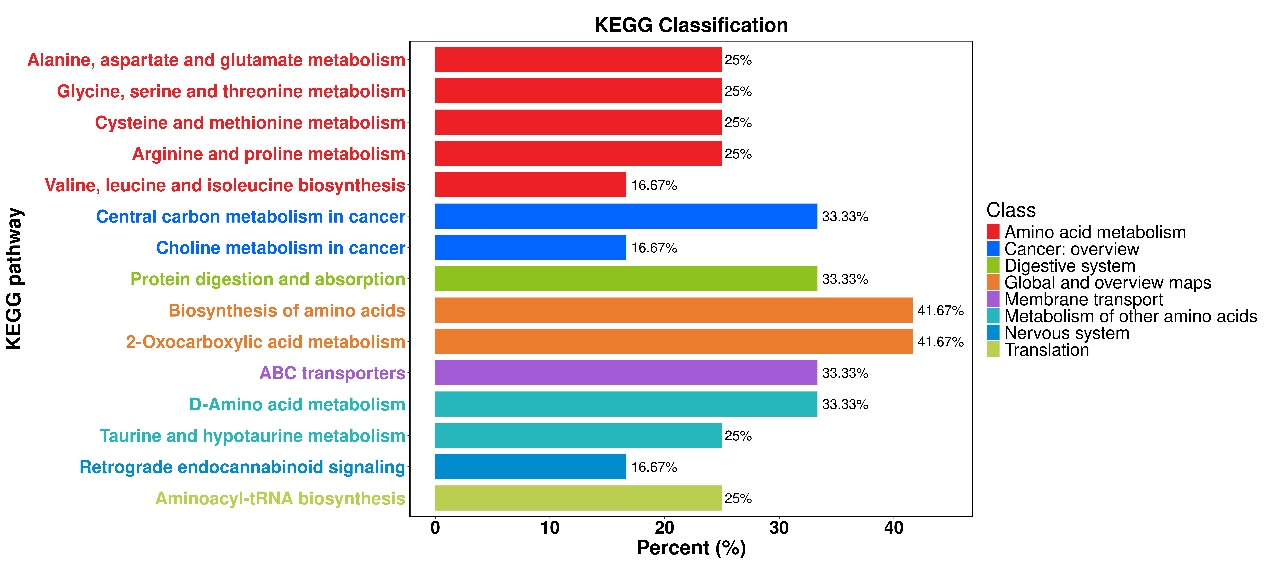


**Figure S9. KEGG Annotation Analysis of Differential Metabolites among the three groups.**

Note. The x-axis represents the proportion of enriched metabolites per KEGG pathway, while the y-axis lists pathway names. The color legend categorizes pathways by major biological categories (e.g., Metabolism, Signal Transduction) based on KEGG classification.


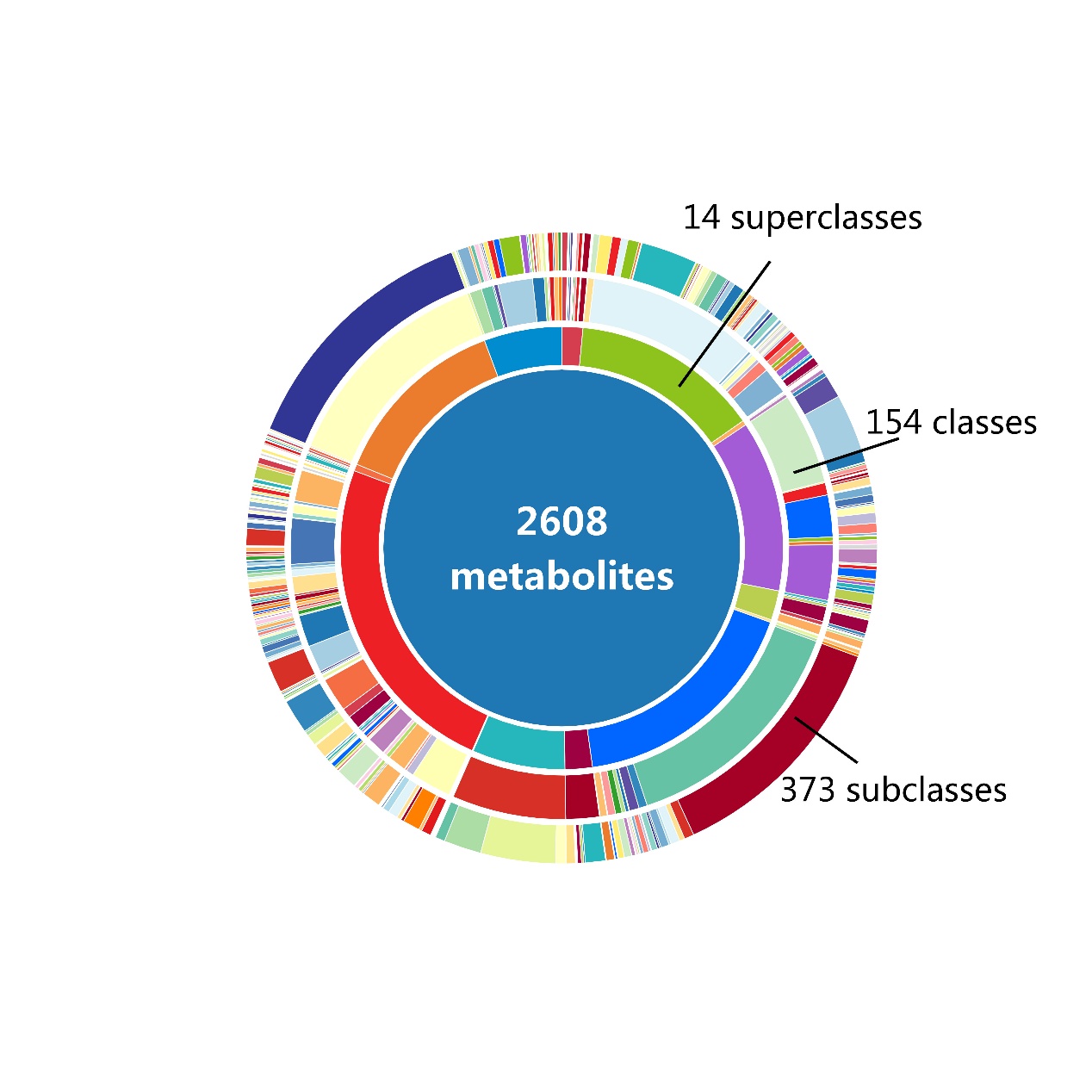


**Figure S10. The circular chart of metabolite classification.**


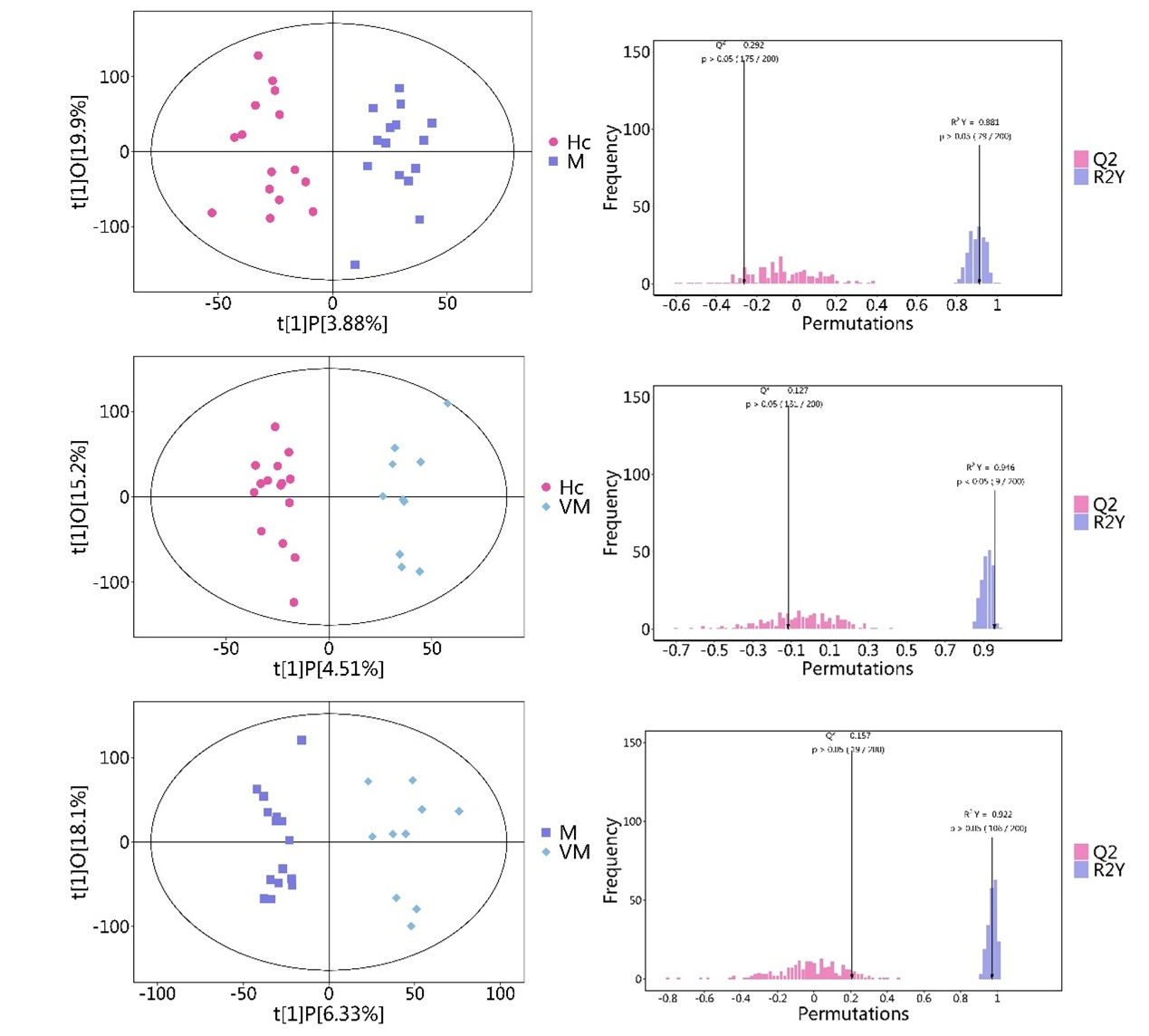


**Figure S11. Scatter plots of OPLS - DA model scores for pairwise comparisons among the three groups (left) and histogram of permutation test results of the OPLS - DA model (right).**

The red bars represent the number of occurrences of Q2 values obtained from the permutation test, and the blue bars represent the number of occurrences of R2Y values obtained from the permutation test. The p value = the number of random models in the permutation test that are superior to the original model / the total number of random models in the permutation test. Generally, when P<0.05, the model is considered optimal.


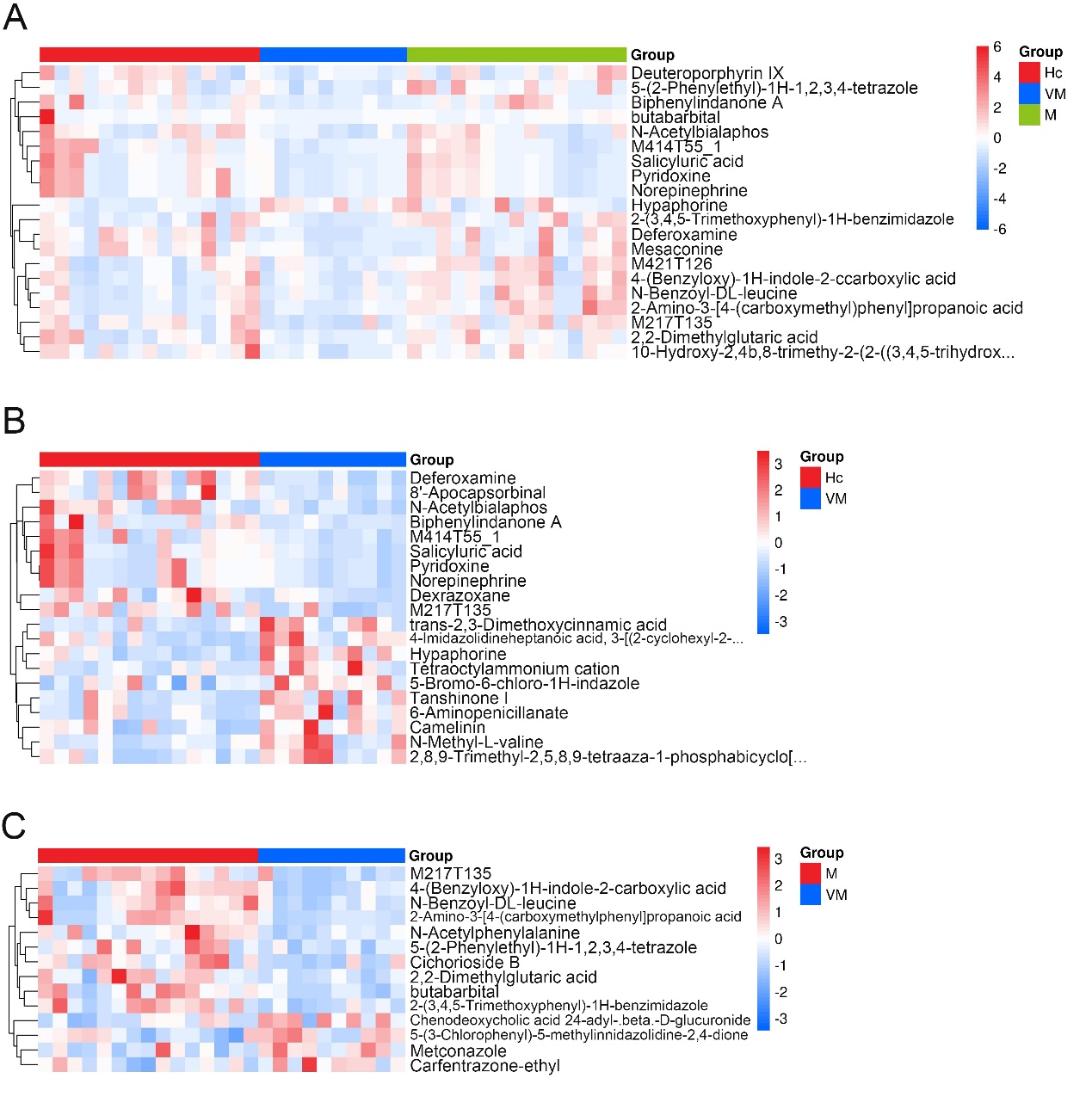


**Figure S12. The top 20 metabolites with significant differences.**

A. The top 20 metabolites with significant differences among the three groups.

B. The top 20 metabolites with significant differences between the VM and Hc groups.

C. The top 20 metabolites with significant differences between the VM and M groups.


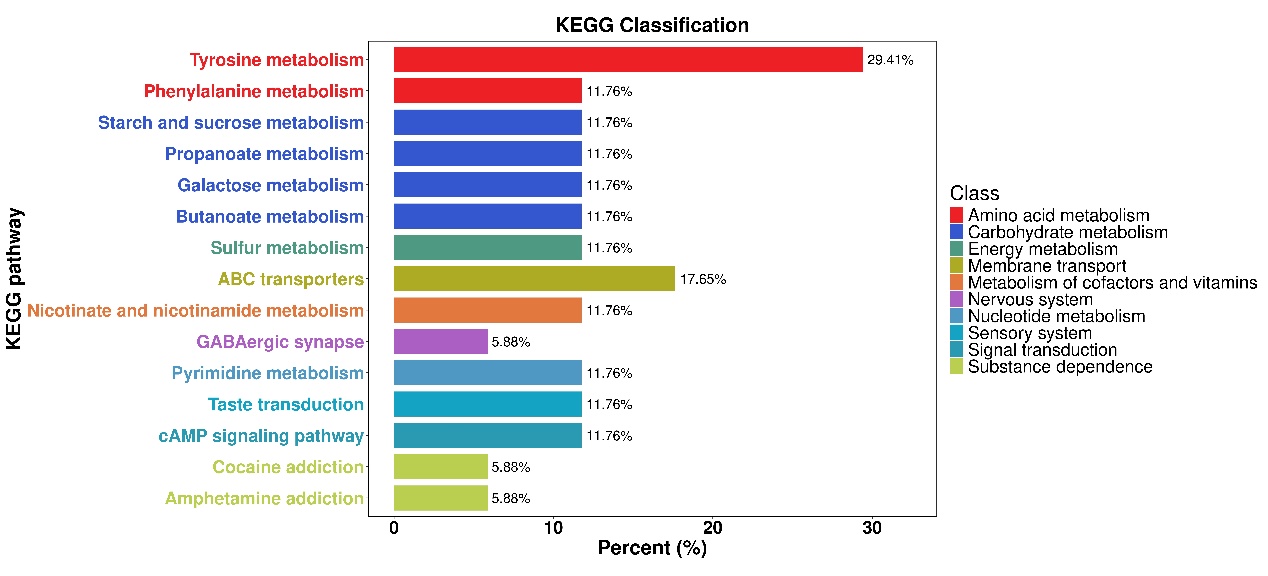


**Figure S13. KEGG Annotation Analysis of Differential Metabolites among the three groups.**

Note. The x-axis represents the proportion of enriched metabolites per KEGG pathway, while the y-axis lists pathway names. The color legend categorizes pathways by major biological categories based on KEGG classification.


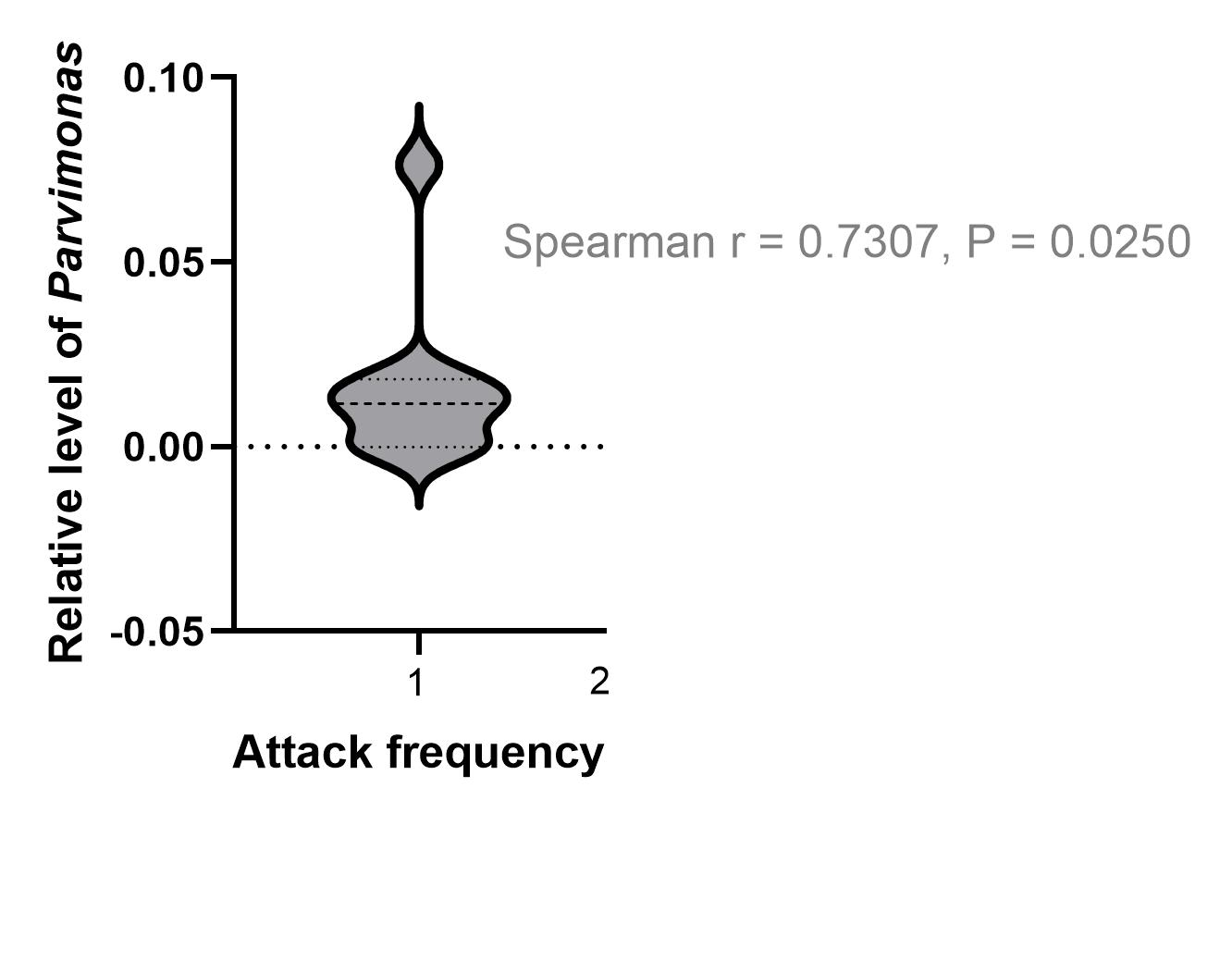


**Figure S14A. Correlation between the relative abundance of *Parvimonas* and attack frequency.**


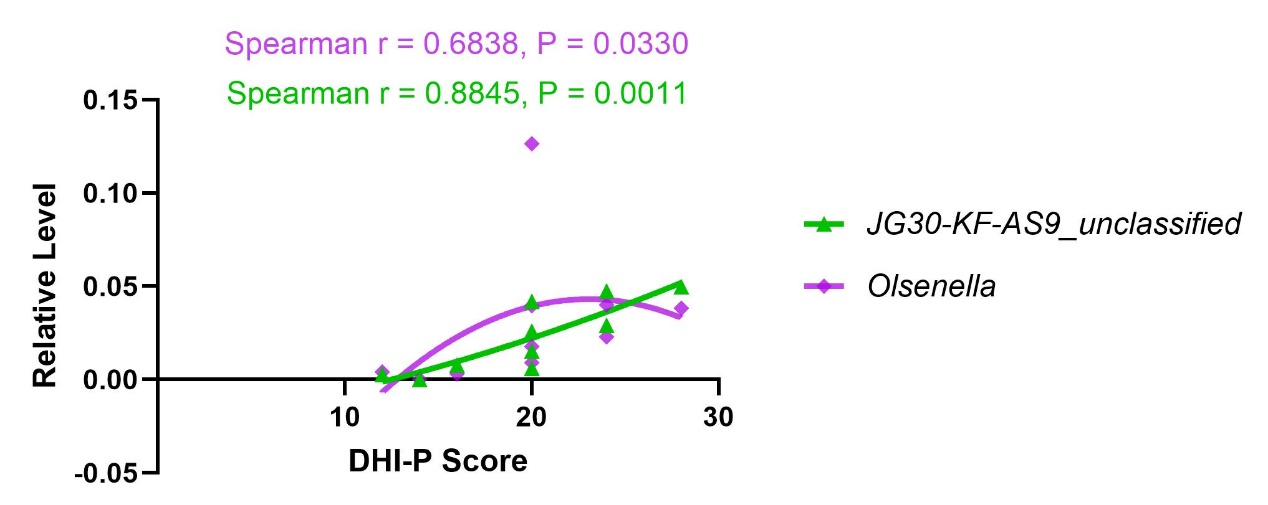


**Figure S14B. Correlation between the relative abundance of *JG30-KF-AS9_unclassidied*, *Olsenella* and DHI-P score.**


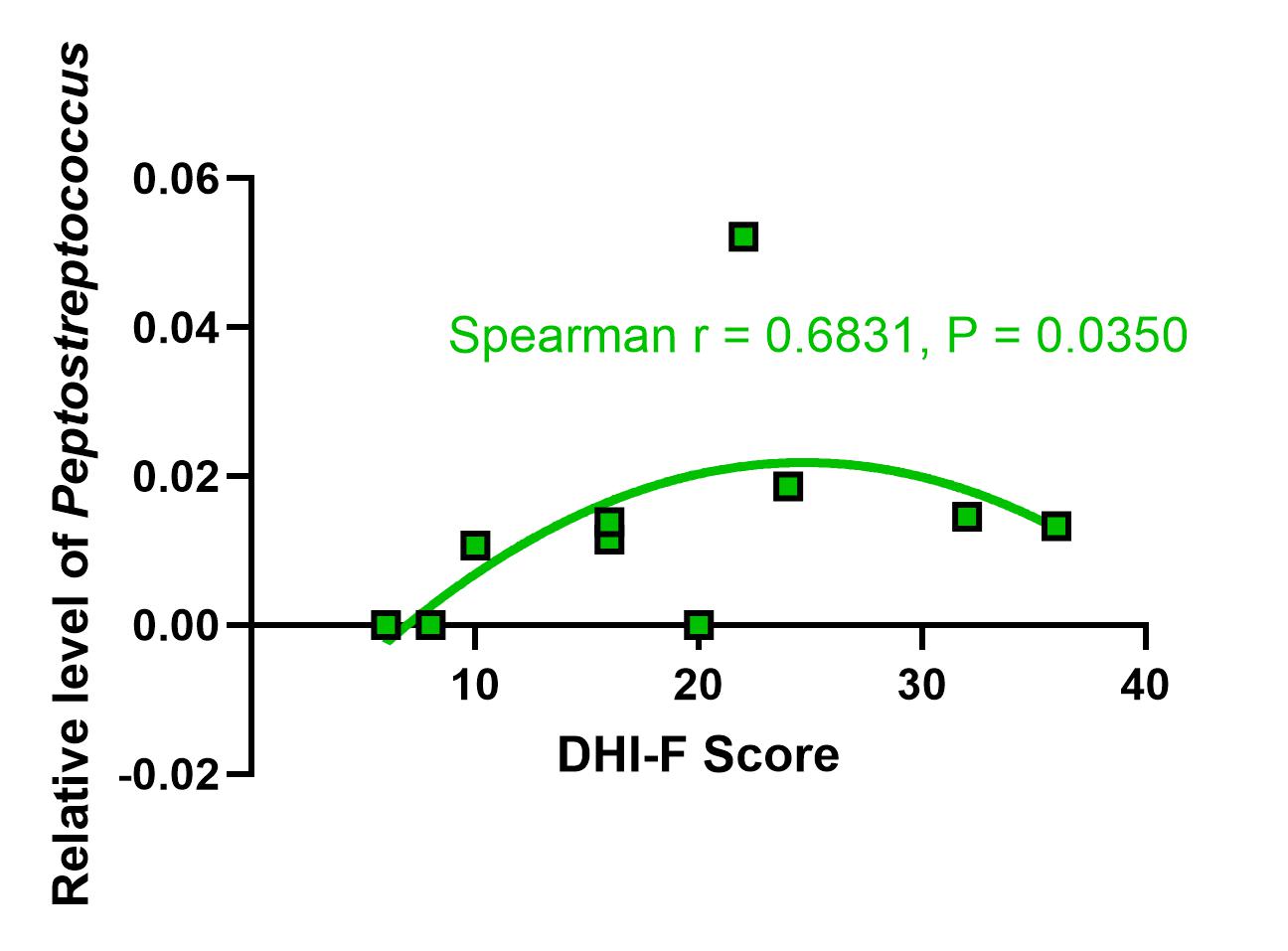


**Figure S14C. Correlation between the relative abundance of *Peptostreptococcus* and DHI-F score.**


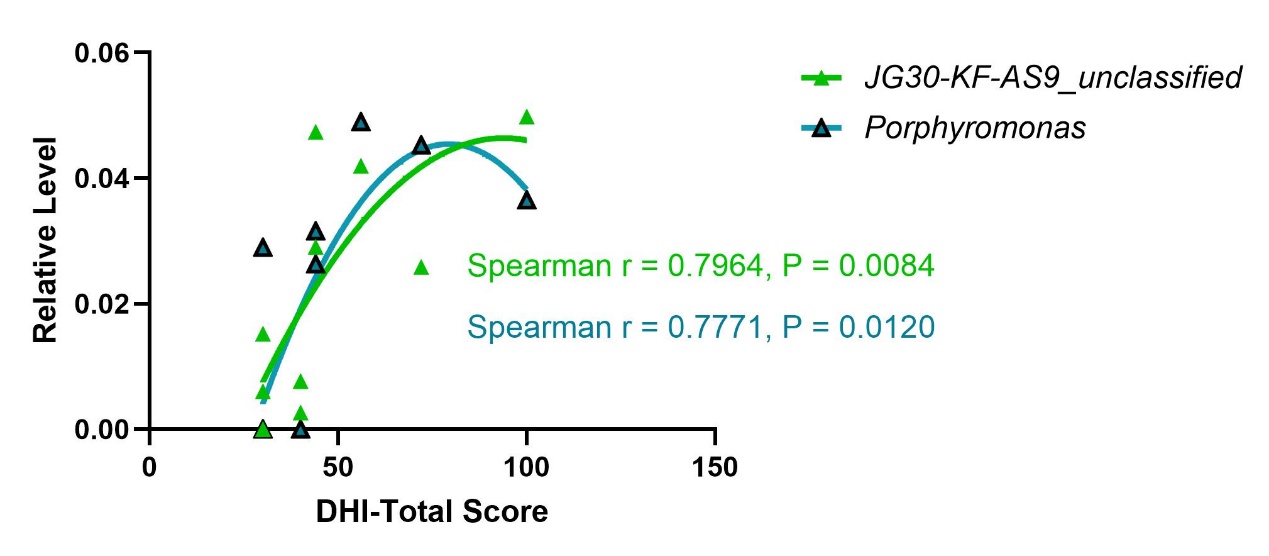


**Figure S14D. Correlation between the relative abundance of *JG30-KF-AS9_unclassidied*, *Porphyromonas* and DHI-Total score.**


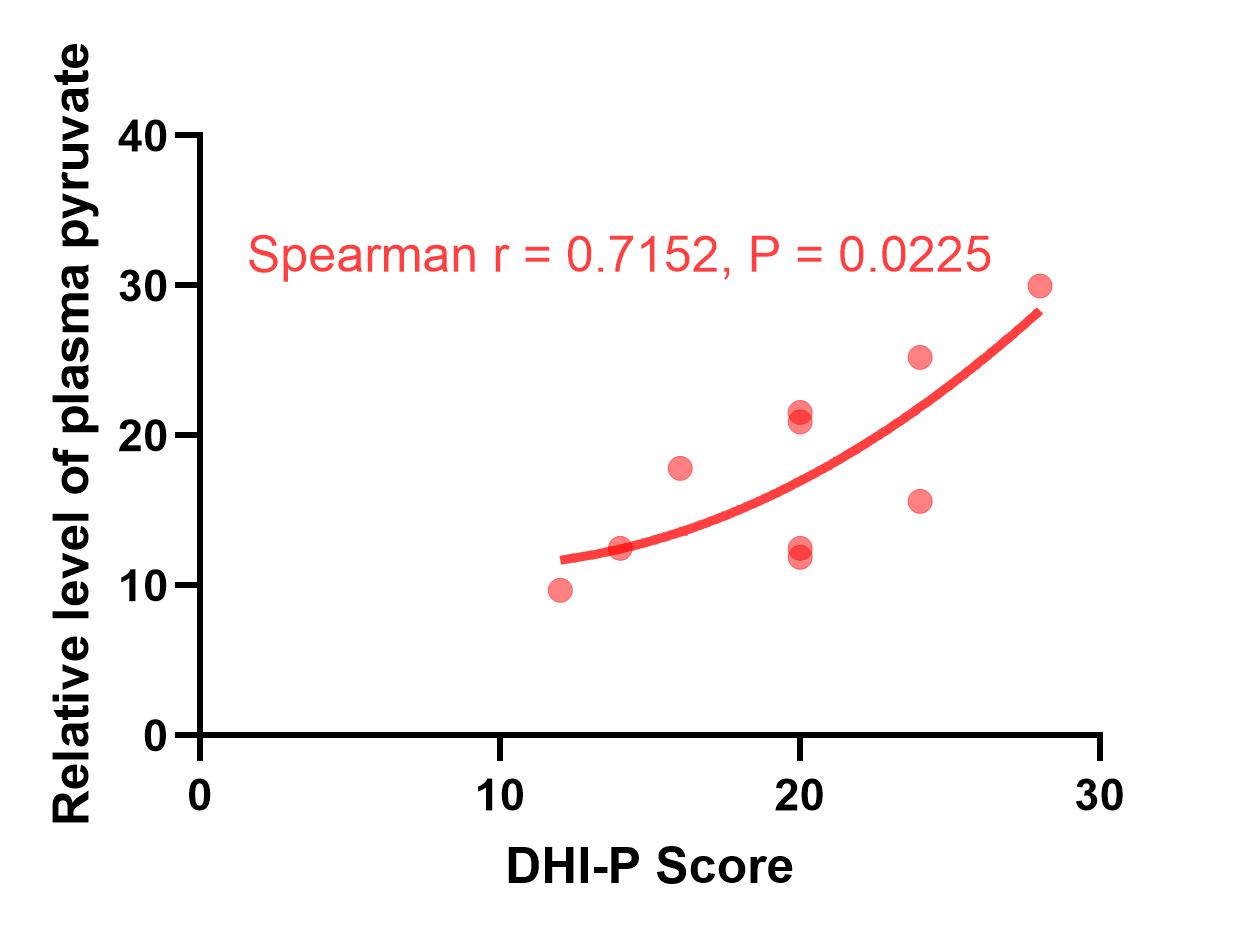


**Figure S15. Correlation between the relative abundance of plasma pyruvate and DHI-P score.**


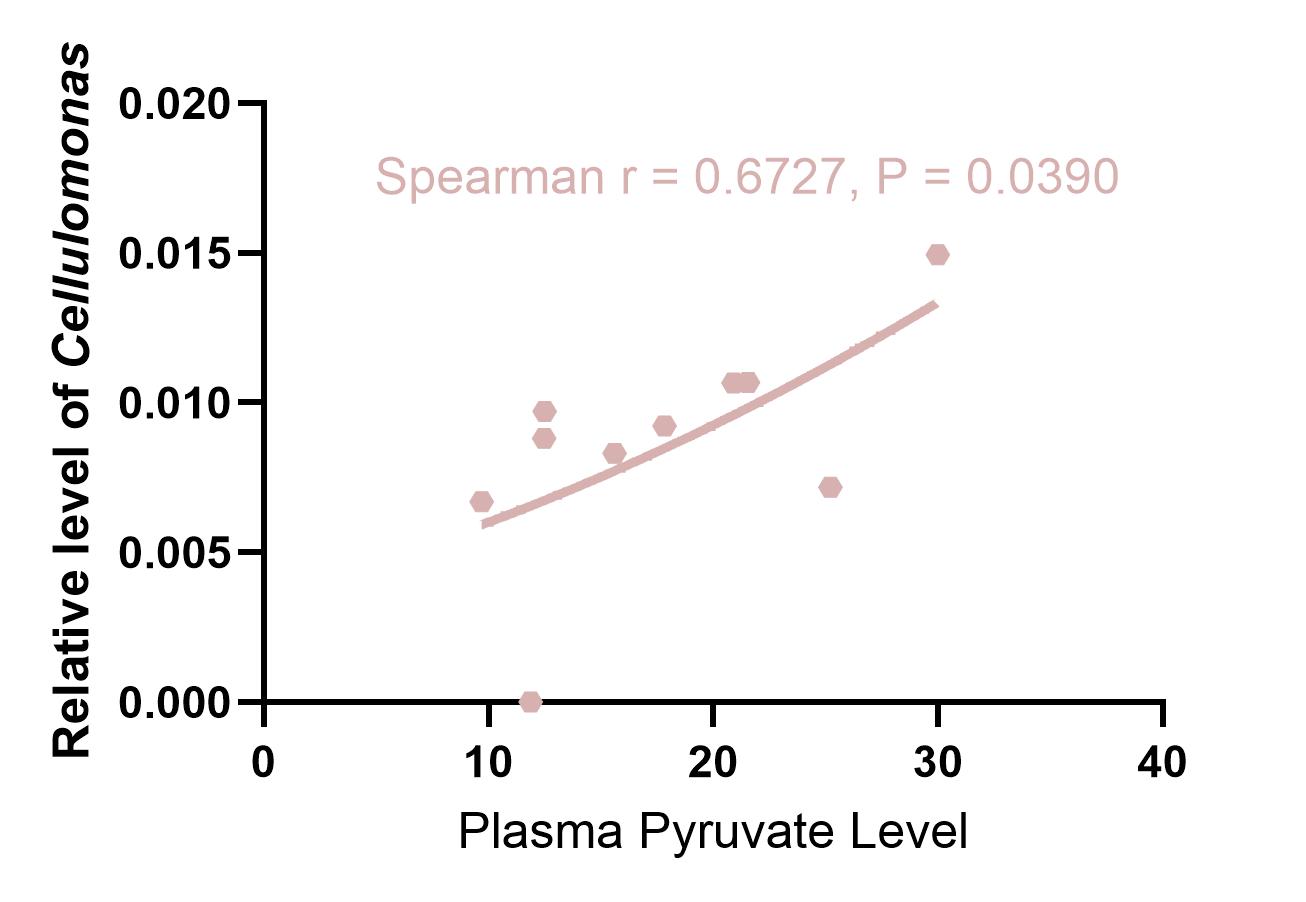


**Figure S16. Correlation between the relative abundance of Cellulomonas and plasma pyruvate.**

**Table S1. The results of Chao1, observed_ species, Shannon, Simpson,** **pielou-e and Goods _coverage**

| Alpha diversity | group1 | group2 | P | Significance |
| --- | --- | --- | --- | --- |
| observed_species | Hc | M | 0.198 | ns |
| observed_species | Hc | VM | 0.233 | ns |
| observed_species | M | VM | 0.048 | * |
| shannon | Hc | M | 0.713 | ns |
| shannon | Hc | VM | 0.495 | ns |
| shannon | M | VM | 0.238 | ns |
| simpson | Hc | M | 0.967 | ns |
| simpson | Hc | VM | 0.531 | ns |
| simpson | M | VM | 0.367 | ns |
| chao1 | Hc | M | 0.250 | ns |
| chao1 | Hc | VM | 0.261 | * |
| chao1 | M | VM | 0.041 | * |
| goods_coverage | Hc | M | 0.442 | ns |
| goods_coverage | Hc | VM | 0.389 | ns |
| goods_coverage | M | VM | 0.134 | ns |
| pielou_e | Hc | M | 0.838 | ns |
| pielou_e | Hc | VM | 0.723 | ns |
| pielou_e | M | VM | 0.367 | ns |
| ace | Hc | M | 0.250 | ns |
| ace | Hc | VM | 0.261 | ns |
| ace | M | VM | 0.041 | * |

Note. “ns” means no statistical significance, “*” means statistical significance.

## Table S2. The number of differential metabolites among the three groups (including up-regulated and down-regulated ones).

| Comparison | Cpd_all | Cpd_diff | Cpd_diff_up | Cpd_diff_down |
| --- | --- | --- | --- | --- |
| Hc -M | 1157 | 47 | 11 | 36 |
| Hc-VM | 1157 | 54 | 46 | 8 |
| M-VM | 1157 | 115 | 102 | 13 |

Note. “Cpd_all” represents the total number of detected substances, “Cpd_diff” represents the number of substances with differential expression in the group comparison, and “Cpd_diff_up” and “Cpd_diff_down” represent the numbers of up-regulated and down-regulated differential substances in the group comparison, respectively.

**Table S3. The number of differential metabolites among the three groups (including up-regulated and down-regulated ones).**

| Group | Cpd_all | Cpd_diff | Cpd_diff_up | Cpd_diff_down |
| --- | --- | --- | --- | --- |
| Hc-M | 2608 | 62 | 54 | 8 |
| Hc-VM | 2608 | 51 | 11 | 40 |
| M-VM | 2608 | 165 | 4 | 161 |

Note. “Cpd_all” represents the total number of detected substances, “Cpd_diff” represents the number of substances with differential expression in the group comparison, and “Cpd_diff_up” and “Cpd_diff_down” represent the numbers of up-regulated and down-regulated differential substances in the group comparison, respectively.

**Table S4. Differential metabolites mapped to significantly enriched pathways in KEGG topological analysis across comparative groups**

| Comparison | Pathways | Differential metabolites |
| --- | --- | --- |
| Hc-M-VM | Alanine, aspartate and glutamate metabolism | **Pyruvate**, L-glutamate |
| Hc-VM | Alanine, aspartate and glutamate metabolism | **Pyruvate**, L-glutamate |
| M-VM | Ubiquinone and other terpenoid-quinone biosynthesis | ​（3-(4-Hydroxyphenyl)lactate）, Alpha-Tocopherol |

Note: Bold entries belong to the top 20 significant differential metabolites mapped to the pathways in specific inter-group comparison.
